# Translating Akkadian to English with neural machine translation

**DOI:** 10.1093/pnasnexus/pgad096

**Published:** 2023-05-02

**Authors:** Gai Gutherz, Shai Gordin, Luis Sáenz, Omer Levy, Jonathan Berant

**Affiliations:** School of Computer Sciences, Tel Aviv University, Tel Aviv 69978, Israel; Digital Pasts Lab, Department of Land of Israel Studies and Archaeology, Ariel University, Ariel 40700, Israel; Digital Pasts Lab, Department of Land of Israel Studies and Archaeology, Ariel University, Ariel 40700, Israel; Seminar für Sprachen und Kulturen des Vorderen Orients, Heidelberg University, Heidelberg 69117, Germany; School of Computer Sciences, Tel Aviv University, Tel Aviv 69978, Israel; School of Computer Sciences, Tel Aviv University, Tel Aviv 69978, Israel

**Keywords:** Babylonian heritage, cuneiform script, ancient low-resource language, Akkadian–English translation, machine translation, neural networks

## Abstract

Cuneiform is one of the earliest writing systems in recorded human history (ca. 3,400 BCE–75 CE). Hundreds of thousands of such texts were found over the last two centuries, most of which are written in Sumerian and Akkadian. We show the high potential in assisting scholars and interested laypeople alike, by using natural language processing (NLP) methods such as convolutional neural networks (CNN), to automatically translate Akkadian from cuneiform Unicode glyphs directly to English (C2E) and from transliteration to English (T2E). We show that high-quality translations can be obtained when translating directly from cuneiform to English, as we get 36.52 and 37.47 Best Bilingual Evaluation Understudy 4 (BLEU4) scores for C2E and T2E, respectively. For C2E, our model is better than the translation memory baseline in 9.43, and for T2E, the difference is even higher and stands at 13.96. The model achieves best results in short- and medium-length sentences (c. 118 or less characters). As the number of digitized texts grows, the model can be improved by further training as part of a human-in-the-loop system which corrects the results.

**Significance Statement**

Hundreds of thousands of clay tablets inscribed in the cuneiform script document the political, social, economic, and scientific history of ancient Mesopotamia. Yet, most of these documents remain untranslated and inaccessible due to their sheer number and limited quantity of experts able to read them. This paper presents a state of the art neural machine translation model for the automatic translation of Akkadian texts into English, from Unicode cuneiform glyphs and from transliterations of the cuneiform signs, achieving 36.52 and 37.47 Best Bilingual Evaluation Understudy 4 (BLEU4) scores, respectively. It is particularly effective in maintaining the style of the text genre in the translation. This is another major step toward the preservation and dissemination of the cultural heritage of ancient Mesopotamia.

## Introduction

Translation is a fundamental human activity, with a long scholarly history since the beginning of writing (see *Materials and methods*). It can be a complex process, since it commonly requires not only expert knowledge of 2 different languages, but also of different cultural milieus. Digital tools that can assist with translation are becoming more ubiquitous every year, tied to advances in fields like optical character recognition (OCR) and machine translation (1, 2). Ancient languages, however, still pose a towering problem in this regard. Their reading and comprehension requires knowledge of a long dead linguistic community, and moreover, the texts themselves can also be very fragmentary. In this paper, we present the first neural machine translation (NMT) into English from Akkadian, one of the oldest yet better attested ancient languages (ca. 2,700 BCE–75 CE). The goal of an NMT system for Akkadian is to be part of a human–machine collaboration, by creating a pipeline that assists the scholar or student of the ancient language. Currently, the NMT model is available on an online notebook and our source code can be found on GitHub at Akkademia. To make our pipeline widely accessible, we are implementing its functionalities into an online application called the Babylonian Engine. These functionalities are especially aimed at analyzing cuneiform texts with computational methods.

**Fig. 1.**
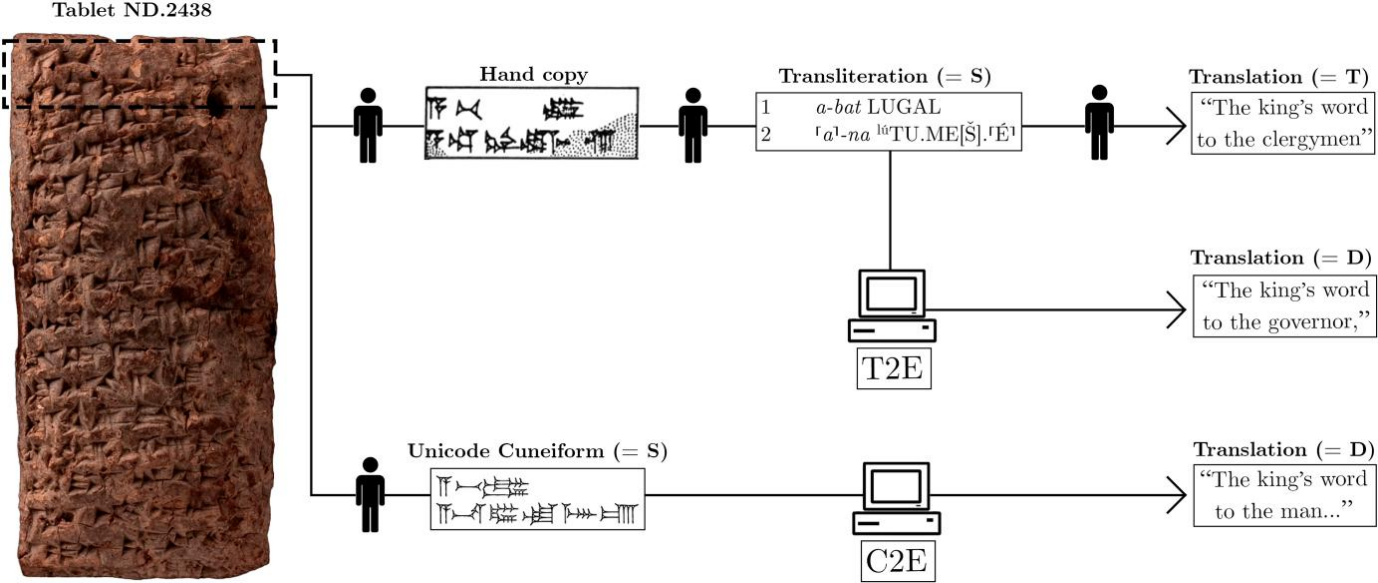
A schematic overview of the NMT model's pipeline. The short designations in parentheses follow the conventions of the test set provided in the SI: the source or input is S, the original HT is T, and the output machine translation is D. Image of the tablet ND.2438 available on the website of the Cuneiform Digital Library Initiative (CDLI: https://cdli.ucla.edu/P393604). © The Trustees of the British Museum.

In this paper, we define translation as the process of taking a source language encoded in its own script and re-encoding it into a target language and script (see also the conventions of the NMT pipeline in Fig. 1). Given that Akkadian is a dead language and its oral form is not recorded, our results are based only on its written form. The resulting translation has to fulfill some criteria of equivalence between the source and target. The source comes from the Assyrian and Babylonian dialects of Akkadian, and the target is modern English. The NMT model, in fact, tackles 2 types of Akkadian sources as input for 2 translation tasks from Akkadian to English (see Fig. 1):

- *Cuneiform to English Task* (C2E) processes Unicode cuneiform glyphs—the computational equivalent of the ancient cuneiform signs used to write Akkadian on clay tablets.^1^
- *Transliteration to English Task* (T2E) processes the transliteration of cuneiform in Latin script, which is the commonly used representation of cuneiform signs by experts in scholarly editions of Akkadian texts.

**Fig. 2.**
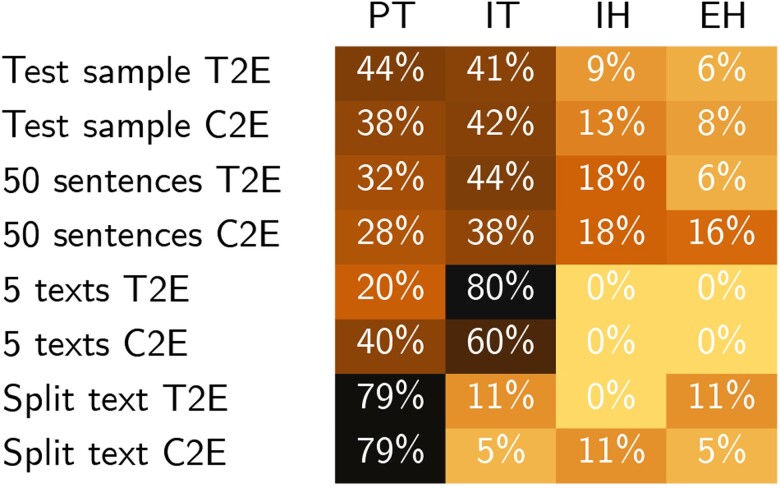
Heat map of the results of the samplings and tests. The percentages indicate how many types of translations are included in each data set. PT, proper translation; IT, improper translation; IH, intrinsic hallucination; and EH, extrinsic hallucination.

Our best results were achieved in the T2E task, reaching 37.47 in the Best Bilingual Evaluation Understudy 4 (BLEU4) score. For comparison, the translation memory baseline achieved a BLEU4 score of 23.51. Overall, however, there was a marginal difference between T2E and C2E tasks (see Table 3). This is promising when translating directly from Unicode cuneiform, which can already be produced by OCR, like the one available for hand copies in the Cuneiform Recognition (CuRe) toolset (see at https://www.ben-digpasts.com/demo). When comparing the model's results to human translation (HT) in our tests (see Fig. 2), the best NMT was produced when sentences were equal or less than the median sentence of the corpus, i.e. 118 characters. An unexpected achievement of both tasks is the reproduction of the input text style and genre.

### Challenges in translating Akkadian

**Fig. 3.**
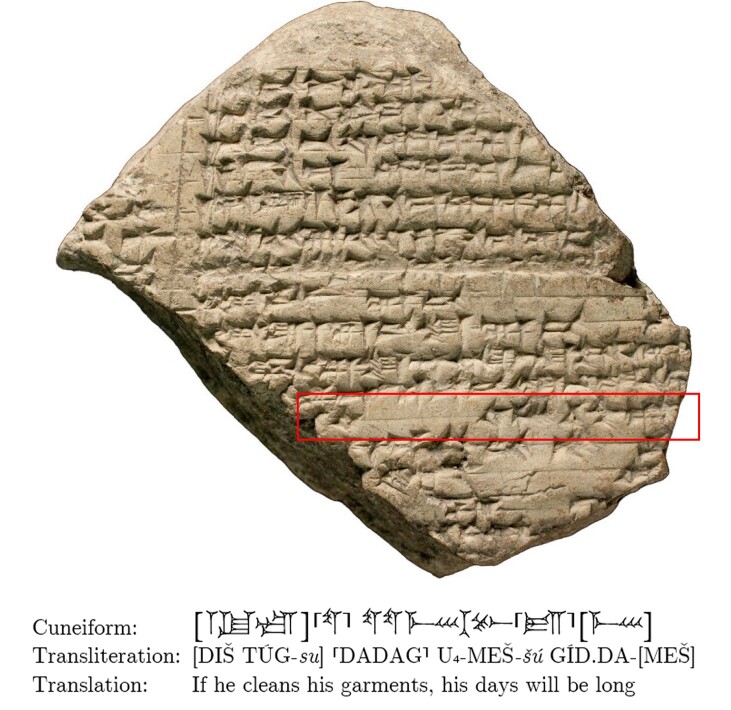
Reverse of the tablet K.8737 (https://cdli.ucla.edu/P238789) from the Library of Ashurbanipal with omens from the series *Iqqur Ippuš* (26). Line 13’ (in red) on the second column contains the logograms DADAG and U_4_, which are written in cuneiform 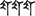 “UD UD UD.” © The Trustees of the British Museum.

There are several challenges in translating an ancient language like Akkadian. Clay tablets are rarely completely preserved. As a result, NMT, as well as HT, are affected by the lack of context. Another challenge is the complex logophonetic nature of cuneiform, i.e. signs can have 1 of 3 functions: logograms, determinatives, and phonograms/syllabograms. Therefore, cuneiform signs are polyvalent and have several readings for each function (see *Materials and methods*). For example, the sign 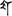 “UD,” originally a pictograph of the Sun(-god), has more than 17 phonetic and 6 logographic values that can only be securely read in context (see Fig. 3). Sometimes, even experts cannot figure out the proper sign value (see Fig. S1).

Experts do not translate directly to a modern language from the ancient cuneiform signs. They first gauge the context of each sign in a given sequence—a process which is called transliteration, resulting in a transcription of the cuneiform signs in the Latin alphabet. Thus, HT has 2 steps: first transliteration and only then translation. The first step of transliteration is considered in computational terms to be another sort of translation task. It can include word segmentation, as cuneiform practically lacks any sort of punctuation marks. We have successfully dealt with this task in a previous study, which resulted in 97% accuracy in transliterating Unicode cuneiform glyphs of Neo-Assyrian texts (3).

Therefore, C2E is considered a more complex translation task than T2E. C2E requires translation between 2 different scripts, Unicode cuneiform glyphs to Latin script, as well as identification of word segmentation. The act of transliteration not only removes the levels of sign and word ambiguity from the cuneiform signs, it also simplifies the task of translation between the same script type; both source and HT are in Latin characters. Interestingly, though we anticipated the results of T2E to be better, there was little to no substantial degradation of the results compared with C2E.

Another issue to consider when translating Akkadian is the different styles of each text genre. The more formulaic the genre of the source, the more accurate the translation will be. Generally, administrative and divinatory texts tend to be very formulaic. We expect the effectiveness of the NMT model to be determined also by the number of texts from each genre used in the training data set (see Table 1). Furthermore, texts in Akkadian are rarely written entirely syllabically, as described above, and almost all of them use Sumerian logograms (transliterated in capital letters). Certain genres, such as divination literature shown in Fig. 3, use logograms profusely, with many sentences made up only of logograms. Often, the names of people, places, and temples are themselves complex sentences written using logograms (see Fig. S1). Thus, our NMT model may interpret them not as proper names but will try to provide a translation of their meaning.

**Table 1.** Corpora used for training with the number of attested tablets arranged according to genres. For the text typologies, see the Archival Texts of the Assyrian Empire (ATAE) project page. See also the typology in the CDLI wiki: https://cdli.ox.ac.uk/wiki/doku.php?id=text_typologies.

Corpus	Genre	Quantity
RINAP	Royal inscriptions	1,794
RIAo	Royal inscriptions	886
RIBo	Royal inscriptions	284
SAAo		(5,059)
	Administrative letters	2003
	Legal transactions	829
	Astrological reports	567
	Administrative records	453
	Scholarly letters	389
	Extispicy queries	278
	Priestly letters	210
	Extispicy reports	76
	Grants	67
	Royal rituals	55
	Literary works	52
	Eponym lists	23
	Treaties	15
	Decrees	14
	Votive donations	12
	Prophecies	11
	Appointments	4
	Gifts	1
Suhu	Royal inscriptions	33
**Total**		**8,056**

### The digital corpora

For this research, we used the following corpora from the Open Richly Annotated Cuneiform Corpus (ORACC), which include transliterations and their equivalent English translations: RINAP, RIAo, RIBo, SAAo, and Suhu (for a more detailed description of the corpora, see Table S3 in the SI).^2^

Chronologically, the great majority of the texts are Neo-Assyrian (see Table 2), and the best attested genres are the royal inscriptions (2,997) and administrative letters (2,003). Nevertheless, the chosen corpus as a whole represents a variety of genres as detailed in their breakdown presented in Table 1. For T2E we used 56,160 sentences, where we treat each sentence as an independent example for training. We call them in this article “sentences,” even if it is a single word, a group of words, a phrase, or a group of phrases. The shortest sentences in the C2E, for example, are made up of 3 characters, while the longest is 237 characters long. The median, 118 characters, is considered a medium-length sentence. This is relevant because, as stated later in the human evaluation, the length of a sentence has an effect on the NMT's performance (see subsection *Human evaluation of NMT* on page 4).

**Table 2.** Corpora used for training arranged according to time period, with the number of attested tablets (for periodization in ancient Mesopotamia, see CDLI wiki: https://cdli.ox.ac.uk/wiki/doku.php?id=adopted_periodisation_in_cdli).

Period	Quantity
Neo-Assyrian	7,327
Middle Assyrian	294
Neo-Babylonian	289
Old Assyrian	122
Unidentified	13
Neo-Assyrian, Neo-Babylonian	7
Old Akkadian	2
Late Babylonian	1
Ur III	1
**Total**	8,056

We pooled all corpora together and partitioned them in the following manner: 90% for training (50,544 sentences), 5% for validation (2,808 sentences), and 5% for testing (2,808 sentences). The average length of a sentence is 15.68 characters with 3,723 sentences over 50 characters long and 61 over 200 characters long. There are 2,440 unique transliterations and 30,101 unique English words.

For C2E, we built the data set differently, using fewer sentences that are longer: overall 50,299 sentences. The percentage for training, validation, and testing is the same, meaning that we used 90% for training (45,269 sentences), 5% for validation (2,515 sentences), and 5% for testing (2,515 sentences). The average length of a sentence is 17.93 characters with 3,957 sentences over 50 characters long and 64 over 200 characters long. There are 639 unique Cuneiform signs.

All the corpora were divided into sentences after verifying no text appears more than once. Then, we randomly took 90% of sentences for training, 5% for validation, and 5% for testing. In terms of machine translation, this number of cuneiform to English samples in our data set represents a challenging low-resource NMT scenario.

For the purposes of C2E, the signs of each text were encoded as strings of Unicode cuneiform glyphs generated by the *Cuneify* tool. Thus, we could use unsegmented strings of Unicode glyphs as our input, with the output being the English translation. It is important to note that the cuneiform signs used in this study are a digitized Unicode representation of sign values, as opposed to sign forms. The visual identification of the cuneiform syllabary, which will enable identifying signs on original tablets with their Unicode equivalent, remains outside the purview of the current study (for literature on visual cuneiform analysis, see (4)). The OCR tool on the Babylonian Engine web application (https://www.ben-digpasts.com/demo) already allows to produce Unicode glyphs directly from an image of a cuneiform hand copy.

### Data set challenges

The ORACC data set is not segmented into sentences, neither in the Akkadian source nor in the English target. Therefore, lines (“sentences”) in the corpus are long. In addition, the data used have some alignment inaccuracies. The English translation does not correspond to the line division in Akkadian which we used as “sentences.” Furthermore, there are broken segments in the texts, which compound the issue. This can lead to redundant or missing English words corresponding to the source (either cuneiform or transliteration).

In addition, we had to take into consideration the issue of text duplication. We verified we have only one copy of each text, even if it appears multiple times in a corpus. Examples without a proper translation were deleted, i.e. with notes such as “No translation possible,” “No translation warranted,” or “broken for translation.” Examples with restorations were also used.

## Algorithmic background

### Tokenization

Tokenization is the process of partitioning a string of characters into a sequence of symbols, which are then fed as input into the NMT model. A simple approach for tokenization is character-based tokenization, where the string is split into its characters. For the Cuneiform script (C2E), we used character-based tokenization with a small vocabulary of characters (400).

While character-based tokenization is simple, it leads to long sequences that are composed of characters that do not necessarily correspond to semantic units. Thus, a popular approach is to use an unsupervised tokenizer that takes a text corpus as input and outputs a vocabulary of predetermined size that includes words and word parts that are then used to segment the corpus. Typically, common words are included in the output vocabulary, while more rare words are broken into smaller parts. For transliteration (T2E), we used BytePair Encoding (BPE) (5) with the SentencePiece package (6), where the size of the vocabulary for the transliteration and English was set to 1,000 and 10,000, respectively.

### Fairseq convolutional model

To train our NMT model, we used Fairseq (7). Fairseq is a sequence modeling toolkit that allows researchers and developers to train custom models for translation, summarization, language modeling, and other text generation tasks.

We used the convolutional model for translation. A convolutional neural network (CNN) is a type of artificial neural network commonly used in image recognition and processing, which has been shown to work well for low-resource and character-level machine translation (8). Another advantage is its shorter training time compared with the popular transformers (8).

### Experimental setting

We set hyperparameters based on the performance on the validation set and report performance on the test set. The parameters tuned are *archictecture*, fconv; *dropout*, 0.1; *criterion*, label smoothed cross-entropy; *labelsmoothing*, 0.1; *optimizer*, nag; *clip-norm*, 0.1; *lr-scheduler*, fixed; *force-anneal*, 50; *max-tokens*, 4000; and *learning-rate*, 0.1. Tables S1 and S2 present the scores for different hyperparameters.

## Results

We evaluated the performance with BLEU4. BLEU is one of the most popular metrics for comparing a candidate translation of a text to one or more reference translations (9). To calculate BLEU, one takes *n*-grams (segments of length *n*) from the predicted translation and checks if they appear in one of the reference translations. We used BLEU4, where a weight of 25% is given to each of the 1-gram, 2-gram, 3-gram, and 4-gram scores.

We used translation memory as a baseline for comparison. A translation memory is a database that stores sentences, paragraphs, or segments of text that have been translated before. Translation memory can be particularly useful when translating formulaic text. Table 3 shows BLEU4 test results for both C2E and T2E for our model and the translation memory baseline. In the next section, we also report expert evaluation of the outputs of the NMT model. In C2E, the NMT model gets a BLEU4 score higher at 9.43 from the baseline, and in T2E, the difference is even higher and stands at 13.96. This shows that the NMT model performs much better than the translation memory baseline. The BLEU4 scores for C2E and T2E were quite close, meaning that for the purpose of NMT, the step of transliteration is not necessary. High-quality translations can be obtained when translating directly from cuneiform to English. This is also the standard practice in other languages that involve ideographic symbols, such as Chinese and Japanese.

**Table 3.** Best BLEU4 score for C2E and T2E.

Task	C2E	T2E
NMT model	36.52	37.47
Translation memory baseline	27.09	23.51

### Human evaluation of NMT

Expert human evaluation was performed by the same person on the best test results of T2E and C2E, to assess the models’ efficacy for scholarship in real-world scenarios (see full test results in Supplemental Data Sets S1 and S2). Instead of evaluating sentences in the test set individually, we randomly sampled 32 sentences from T2E (Supplemental Data Set S3) and 24 sentences from C2E (Supplemental Data Set S5) for qualitative assessment. This selection was based on salient features that were observed in the NMTs, like their accuracy or error diversity. In order to evaluate the NMT models (T2E and C2E), we established several criteria, based on expert domain knowledge in Akkadian.

**Fig. 4.**
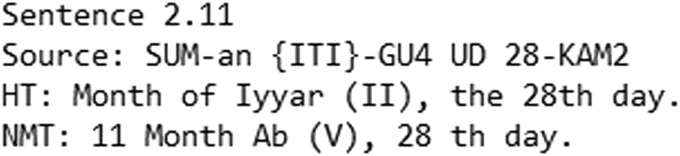
Example of a proper translation from the 5-text test with T2E.

**Fig. 5.**
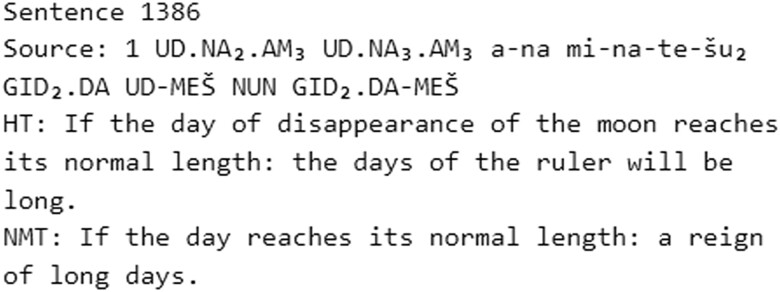
Example of a proper translation from the T2E random sampling.

**Fig. 6.**
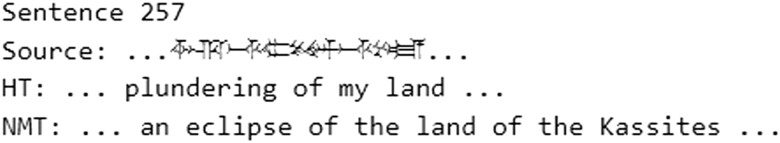
Example of an improper translation from the C2E random sampling.

**Fig. 7.**
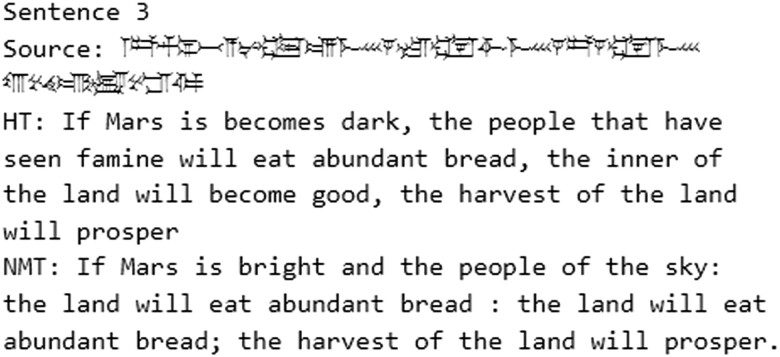
Example of an improper translation from the 5-text test with C2E.

There are 3 types of translations: (i) *proper translation*, the model has produced a usable translation for later refining. It is fluent in the target language and equivalent to the source (see Figs. 4 and 5). This does not imply that the translation is flawless; (ii) *improper translation*, the model has produced an unusable translation, meaning that it is neither fluent in the target language nor equivalent to the source (see Figs. 6 and 7); and (iii) *hallucination*, the model has produced a meaningful translation in the target language, but that meaning is inadequate to the source (10). Sometimes this makes the whole translation unusable as a scholarly translation, but hallucinations also appear inside partly proper NMTs.

**Fig. 8.**
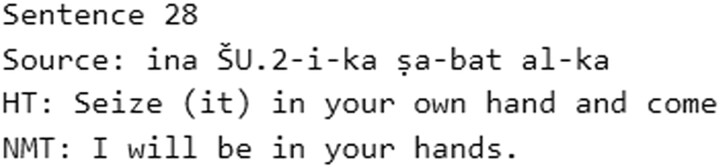
Example of an intrinsic hallucination from the 50-sentence test with T2E.

**Fig. 9.**
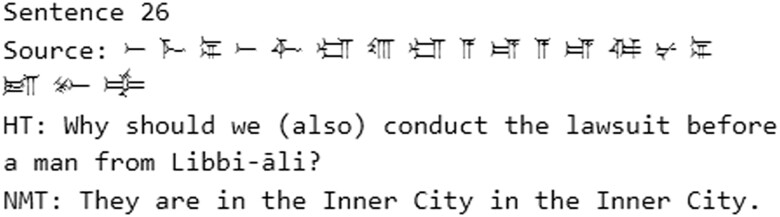
Example of an extrinsic hallucination from the 50-sentence test with C2E.

There are 2 types of hallucinations (11): an *intrinsic hallucination* means that the model has produced a translation error using the input present in the source (see Fig. 8), and an *extrinsic hallucination* means that the model has produced a translation error that ignores the input present in the source. Both types of hallucinations could be semantically and syntactically correct, i.e. a fluent translation, yet always lack equivalence to the source (see Fig. 9).

### T2E random sampling (see Supplemental Data Sets S4 and S5)

Out of 32 sentences randomly sampled from the test set, 14 were properly translated, 5 had interesting hallucinations, and 13 were improperly translated (see Fig. 2).

Sentences 840, 1,237, and 1,386 are good examples of a proper translation. Though the NMT in 1,386 is not the same as the HT, it is very close and definitely useful. Sentences 839 and 2,480 show proper translations, but they are surprisingly more literal than the HT. In the sentence 1,326, the NMT is slightly better than the HT because the sign for the number 1 at the beginning of the line is correctly translated as the conjunction “if” (Akk. *šumma*). The best results are to be found in medium to large sentences (i.e. more than 118 characters in length), like 1,853 and 1,386. In many cases, the NMTs are the same as the HT, like in sentences 618, 1,075, and 1,789.

Cases of improper translation include, for example, sentences 163 and 2,758. Sentence 365 is a good case of an extrinsic hallucination, since most of the translation is semantically and syntactically correct, but it is not equivalent to the source except for the word “house.” Intrinsic hallucinations could be observed when the model had problems managing long phrases and produces NMTs with repetitions, like in sentences 1,541 and 1,799.

An additional problem with long sentences, although it is not frequent, is that the NMT is considerably shorter than the HT. That means that the NMT is not translating everything that is in the source, e.g. in sentences 56, 179, and 216. There are also some extreme cases, in which the NMT does not translate the source at all, like in 495, 1,725, and 2,114. These can be the result of the misalignment of the Akkadian source and the HT in the training data (see *Data set challenges* on page 3). Overall, however, this issue did not seem to have had a serious effect on the NMT results. Otherwise, a higher percentage of improperly translated sentences would exhibit NMTs that are shorter than their source.

### C2E random sampling (see Supplemental Data Sets S5 and S6)

Out of 24 sentences randomly sampled from the test set for this translation task, 9 were properly translated, 5 exhibited hallucinations, and 10 were improperly translated (see Fig. 2).

Examples of a proper translation are sentences 457, 745, and 1,157. In the case of the latter, an omen, the model adds the word “variant.” That is not in the Akkadian source, but such scholarly additions are common in ancient texts and therefore appear in our training data. In 745, the second personal name in the source is not translated correctly, but the NMT is otherwise a proper translation. In other cases, like sentences 2,552 and 2,553, names are perfectly translated. Generally speaking, in comparison to the T2E test results, the C2E test contains more cases where the NMT is the same as the HT, like in the sampled sentences 1,187 and 1,984. These examples fit well with the general formulaic nature of many Akkadian genres.

Instances of improper translation in the sample include, for example, 108, 257, 1,665 and 1,994. Otherwise, the C2E sample has more examples of hallucinations than the T2E sample. Though this was not tested quantitatively, it would suggest a general trend in the C2E test results. In sentence 1992, for instance, the transliteration of the sentence is *pa-ra-si*, which means “to decide.” The NMT produces the translation: “the face.” This translation is an extrinsic hallucination, possibly based on the reading of the first sign 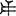 (PA), which is used in other cases to write *ina pa-ni-šu_2_* “in his presence,” literally “in his face.” In sentence 2,494, the NMT produced an extrinsic hallucination, in which the Akkadian word for “lead” 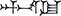
*an-na-ku* was translated as the pronoun “I,” also attested with the same spelling.^3^ It is possible the NMT chose “I” over “lead,” given the more common appearance of the former in Akkadian texts.

### Translation test: human vs machine

In this subsection, we present 2 tests of human vs machine translation. For the *first one*, we chose 50 sentences of different lengths from texts published by cuneiform experts (see Supplemental Data Set S15). In some cases, a whole text was split into sentences. Their genres are distributed according to the 5 best attested genres in the training corpus (see Table S3). For the *second test*, we chose 5 texts (see Supplemental Data Set S16): 3 unpublished, and 2 legal transactions published only with a German translation. The texts were chosen from the Fragmentarium text collection of the eBL project (https://www.ebl.lmu.de/). The 2 legal transactions were each treated as a single long sentence. We also made a second experiment for text 2, in which it was split into smaller sentences which correspond to the division of text lines in the original document (2.1–2.19). This was done to compare the results between long and short sentences in the NMT model (for the definition of long and short, see the subsection *The digital corpora* on page 2). The 3 unpublished texts were translated by 2 cuneiform experts in our team without disagreement and include 1 astrological omen (divination text), 1 literary text, and 1 unknown. All the tests were conducted with both T2E and C2E models.

### Fifty-sentence test with T2E (see Supplemental Data Sets S7–S8)

The results of the 50-sentence test with T2E achieve 16 proper translations, 12 cases of hallucinations, and 22 improper translations (see Fig. 2). The model achieved proper translations in every genre. There are 4 instances, sentences 2, 8, 18 and 34, in which the NMTs are as good as a HT. In other instances, like 14 and 32, the NMT missed only 1 word. We also find useful cases of translation, even in NMTs which exhibit hallucinations or partly improperly translated phrases, for example, intrinsic hallucinations in the form of repetitions inside a proper translation (sentence 35) or a proper translation that only a small part of its source was not translated (sentence 17).

In the improperly translated sentences, there are intrinsic hallucinations in the NMTs in the form of repetitions, like in 7 and 15. This was already observed in the T2E random sample test. Personal names were not recognized as such in numerous cases. Two instances, 16 and 45, are Old Babylonian sentences, which represent a difficulty, as the language as well as the orthography differs from that of later periods on which the model was trained. The NMTs are not proper, but at least the style of the genres is recognizable. This can be useful for the classification of different or varied corpora of texts (see further the *Conclusion*).

### Fifty-sentence test with C2E (see Supplemental Data Sets S9–S10)

The results of the 50-sentence test with the C2E achieve 14 proper translations, 18 cases of hallucinations, and 22 improper translations (see Fig. 2). Like in the previous T2E test, the model achieved proper translations in every genre. There are 3 sentences: 8, 34, and 43, in which the NMTs are as good as a HT. In other instances, like 14, 22, 32, and 38, the NMT mistranslated only 1 word for each sentence. There are also cases where part of the source was not translated at all, e.g. 13, but the NMTs are still useful.

In this test, we also find intrinsic hallucinations in the form of repetitions, like in sentence 7. A more extreme problem is cases in which the NMT does not provide translation for most of the source, like in sentence 1. As observed in the T2E test, the Old Babylonian sentences, 16 and 45, are not properly translated, but the style of the genres is somewhat reproduced. Personal names are not recognized as such in numerous cases.

### Five-text test with T2E (see Supplemental Data Sets S11 and S12)

The results of this test achieve 1 proper translation and 4 improper translations (see Fig. 2). Within the NMTs, hallucinations are to be found. The NMT of the 2 legal transactions are improper (texts 1 and 2). In both, the NMT translated correctly only the first phrase. The personal names are not recognized. It is worthwhile to mention that the names in the original texts are not entirely preserved. Only the style of the genre is reproduced.

There are interesting cases of hallucinations that can be traced to the training data. The NMT of the astrological omen in text 3 is mixed with vocabulary of extispicy omens. We also find some repetitions here. Text 4 is a badly preserved oracular question, which in terms of its genre is similar to an extispicy query (see Table 1). In this sentence, the model produces repetitions, but correctly translates several words from the source. The colophon, where the king Ashurbanipal presents himself, also has some repetitions in the NMT, but it is still a useful translation. Text 5 is an unknown literary text. It may be a passage from the epic of Erra. The context is very fragmentary, making it difficult to decipher. Even for an expert, this text is difficult. In this case, not even the style of the genre is maintained.

The second experiment in which text 2, 1 of the legal transactions, was split into smaller sentences before NMT proves that the best way to translate a text is by splitting it into shorter sentences. Of the 19 sentences, 14 NMTs are proper.

### Five-text test with C2E (see Supplemental Data Sets S13 and S14)

The results of this test present 2 proper translations and 3 improper translations (see Fig. 2). Within the NMTs, hallucinations are to be found. Like in the T2E test, the NMTs of the 2 legal transactions are improper. The personal names are not recognized as well. Only the style of the genre is reproduced.

On the other hand, the astrological omen in text 3 is a proper translation, even though it has some intrinsic hallucinations in the form of repetitions. The NMT of text 4 is considered proper because the colophon is almost completely translated, although there are also repetitions. As said in the T2E test, text 5 is particularly difficult for machine and human alike.

The second experiment with text 2 shows 15 proper NMTs, which is almost the same result as the T2E test. This confirms that the best way to translate a text is by splitting it into shorter sections.

## Discussion

In quantitative terms, the BLEU4 score shows great promise for the NMT model, both in C2E and T2E tasks. In the following qualitative human evaluation of the test results, we characterized the main deficiencies when using the NMT for scholarly purposes. Note the following illustrative example from the T2E test:

*Sentence 2,753*
Source: UD 21-KAM2 LUGAL ina E2-DINGIR E2-DINGIR la ur-rad
HT: “On the 21st day the king does not go down to the House of God.”
NMT: “On the 21st day the king goes down to the House of God.”

The repetition of E_2_-DINGIR in the source is surely an error that occurred when cleaning the data for training, yet the model produced a proper translation of most of the source. The NMT model did not translate the negation and therefore missed the nuanced meaning of the sentence, the result being that even proper NMTs need to be evaluated by experts.

Other observations based on our sample assessments are (i) the NMT can overcome problems in the source, like repetitions or data inputted incorrectly; (ii) despite misalignment issues in the training data, the model still produces satisfactory and useful results for scholars and students; and (iii) the C2E task is more prone to hallucinations given the polyvalent nature of the cuneiform signs (see *Challenges in translating Akkadian* on page 2).

In the human vs machine translation tests, the results with T2E are slightly better than those with C2E, reflecting the close BLEU4 score of the 2 tasks. The NMTs of some text genres, like royal inscriptions, were closer to HT with T2E than with C2E. This genre is also one of the most dominant in the training data. More specifically, in the 5-text test, omens, which are a formulaic genre, had the best NMT results. The 2 legal transactions were not as good as expected. Nevertheless, in all the cases except the unknown text 5, the genre of the source could be identified from the NMT.

Moreover, the additional test of text 2 inputted with division into short sentences clearly shows that long source texts, i.e. long sentences, should be split to get best results.

## Conclusion

For both NMT tasks from Akkadian to English evaluated in this paper, the best results overall were to be found in the short- and middle-length sentences. Longer sources produced more hallucinations or missing translations in the NMT results. This is promising for the usage in realistic scenarios, since all cuneiform texts are divided into manageable lines on the clay tablet. The number of characters on an inscribed clay tablet can vary from period to period (signs in the Old Babylonian period are bigger than in the Neo-Babylonian period) and from genre to genre. Also, the number of columns by which a tablet is divided will determine the number of characters. Even in a single tablet, the number can vary to fill the space in the line. In a Neo-Assyrian royal inscription for example, a line has on average about 20 characters; a tablet of astrological omens can have lines with more characters. Thus, for future use in a human-in-the-loop system, we would define each text line on the tablet as a unit for translation.

Genres with a technical or formulaic style produced better translations. However, the results overall could benefit from manually aligned training data, especially for identifying personal names, and the addition of more genres overall. In the future, the usage of standard sentence aligners such as the Gale–Church algorithm can be considered as a first step of the process to handle the alignment problem of the data set.

The marginal difference between T2E and C2E tasks exhibits a potential advantage when producing translations from the original text, without the need of transliteration. An option for further research is evaluating a multi-source task, using as source both the transliteration and the Cuneiform, as it may outperform each task separately. The input data for C2E could originate from already existing OCR tools in the Babylonian Engine (CuRe Demo and Decuneify), which would even allow a layperson to produce an NMT with the online notebook accompanying this publication, as well as part of the python package Akkademia. In the near future, this NMT model will be added to the Babylonian Engine online portal. This will allow an iterative fine-tuning of the model's capabilities with validated, expertly curated data, in a human-in-the-loop system.

An important achievement of both tasks is the reproduction of the genre's style. This was neither intended nor the main goal of the research. In almost every instance, whether the NMT is proper or not, the genre is recognizable. This provides a kind of summary of the context, recognizing the main content elements of the Akkadian text. A promising future scenario would have the NMT model show the user a list of sources on which they based their translations, which would also be particularly useful for scholarly purposes.

## Related work

This article is part of our ongoing work on artificial intelligence–based pipeline that deals with the reading of cuneiform texts (3, 13). We have dealt with the task of completing broken passages in Akkadian, primarily of archival and administrative texts, in a previous study, using recurrent neural networks (RNN) to complete words in broken context with up to 95% success rate for the top 10 words (13). Using a million tokens from ORACC, another group of scholars recently completed missing signs with 89% success for the top 5 predictions based on a language model that was pretrained on 104 different languages from Wikipedia (14).

Very few studies have attempted machine translation of the cuneiform corpus. To the best of our knowledge, the results presented here are the first attempt to use NMT for Akkadian. The first NMT results for texts written in Sumerian from the Ur III period (see *Materials and methods*) are based on c. 10,000 transliteration to English translation sentences. They report the best BLEU scores with 2 different NMT models of 20.9 and 21.6 and the worst score with a statistical based model of 8.2 (15). Their best model was an attention LSTM model pretrained on English word embeddings from Wikipedia. The data set and results are available in the *Machine Translation and Automated Analysis of Cuneiform Languages* project on GitHub. Morphological analysis of Akkadian, especially in regard to tasks of linguistic annotation, which provide a good framework for machine translation in general, was done by Sahala *et al*. (12, 16).

## Materials and methods

### The cuneiform script and languages in ancient Mesopotamia

In the south of Mesopotamia, at the end of the fourth millennium BCE, a writing system based on ideograms was invented for bookkeeping. The texts were written on clay, and they contained personal names and lists of goods. Thus, the identification of a language behind these ideograms is very problematic, although it is likely that it was Sumerian (17). The first understandable texts come from Ur and are datable to the twenty-eighth century BCE. They are undoubtedly written in Sumerian. Already in these texts, a Semitic name, very likely an Akkadian name, is attested (18). This shows the coexistence of these 2 language communities from almost the beginning of written Mesopotamian history. Cuneiform together with the Akkadian language spread throughout Mesopotamia and became dominant in the ancient Near East until the first millennium BCE, when they were gradually replaced by the Aramaic language and its alphabetic script (19).

Sumerian, an agglutinative language, is attested from the latter part of the fourth millennium BCE until the end of the cuneiform culture around the transition of the common era, when we even find the so-called Graeco-Babyloniaca, Sumerian Greek transliterations (20). After the middle of the second millennium BCE, however, Sumerian was already a dead language used primarily in written form (18). The Akkadian language became dominant from the middle of the second millennium BCE onward. Nevertheless, Akkadian scholars continued to study Sumerian through grammatical texts, bilingual compositions, lexical lists, and school texts (21). Akkadian itself, a member of the east branch of the Semitic language family, was spoken mainly in the north of Mesopotamia. It is one of the best attested languages in antiquity, comparable with Latin (22). Not only Sumerian and Akkadian used the cuneiform script, but also Hittite, Hurrian, Elamite, Ugaritic, and several other smaller languages around the ancient Near East. The last known written documents in Akkadian date from the first century CE, but as a spoken language, it disappeared centuries before (19).

The cuneiform writing system, from its beginning around 3400 BCE until the end of its use, has about 1,000 signs. Nevertheless, not all these signs were used together at the same period of time. They varied from period to period, genre to genre, etc. Besides, the shape of the signs changed geographically as well as diachronically.

**Fig. 10.**
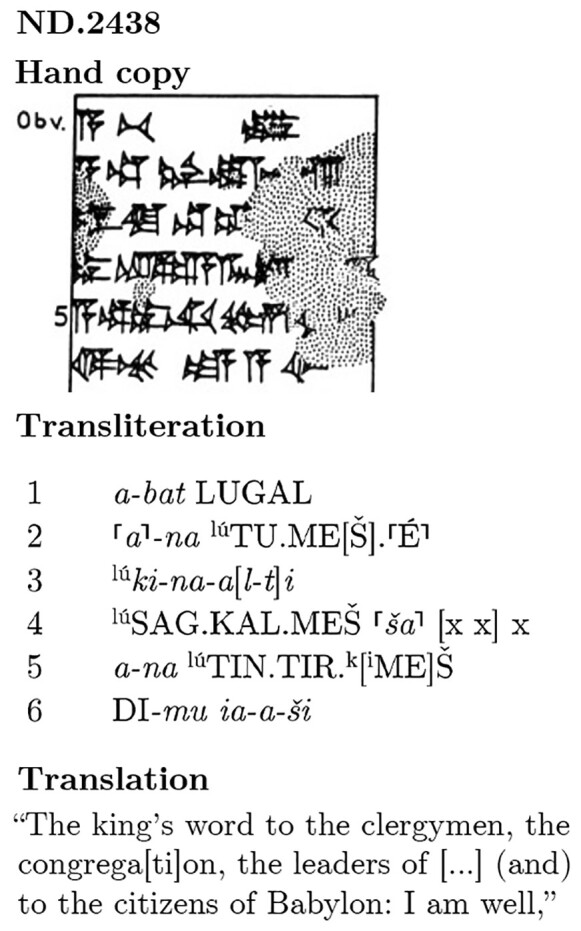
First 6 lines’ edition of the obverse of ND.2438, the same tablet as in Fig. 1. Types of signs according to their function: *italics*, phonograms/syllabograms; CAPITALS, logograms; and ^superscripts^, determinatives. The square brackets indicate a broken sign and the top half brackets, a partially broken sign.

The Mesopotamian cuneiform signs are polyvalent, meaning there is more than one way to read each sign. The Sumerian sign for “well-being,” for example, is written DI (see line 6 in Fig. 10). In this use, it is called a logogram. At the same time, it can be used as a phonetic sign for the sound/di/. Over time, it acquired new phonetic and logographic values, in this case, up to 3 logographic values and 22 phonetic values. This is representative of all cuneiform signs, as they all can have more than one phonetic and logographic value. Three types of functions for the signs are to be found (see Fig. 10):

- Logograms: in most cases designated by capitals in the transliteration (e.g. UTU “sun, bright”), but also in lower case with normal typeface (e.g. utu).
- Determinative: designated in printed transliterations in superscript (e.g. ^d^), but in the digital transliteration, it appears in curly brackets (e.g. {d}).
- Phonograms/syllabograms: depending on the language of the text, they are displayed differently. If the language is Sumerian, they appear in normal script in the transliteration and sometimes in sans serif. If the language is Akkadian, they appear in italics.

The classical editing process usually involves a transcription of cuneiform signs (hand copy), transliteration, translation, and commentary (see Figs. 1 and 10).

### Translation in Mesopotamia

The Mesopotamian tradition of translation has not been well represented in the global history of translation. Most studies begin from Judean, Greek, or Roman sources (23). The coexistence of Sumerian, in the south, and Akkadian, in the north, caused a mutual influence that expresses itself through lexical loans and syntactic changes in both directions. Although it is certain that the contact between these 2 languages predates the inscribed records, translation activities in writing can be traced from the beginning of the second millennium BCE, when Sumerian gradually died as a spoken language and became a language of scholarship and liturgy (18). For the literature of later periods, from the tenth century BCE until the end of the cuneiform script, scholars assume that Akkadian and Sumerian were understood as one and the same language with different codes, as they were fully and mutually translatable (24). In Mesopotamia, all the circumstances that led to translation becoming an academic discipline were present. It possesses not only a long tradition, but also a sophisticated set of tools. As recently emphasized (25), it was also one of the most important activities of Assyrian and Babylonian scholars.

## Supplementary Material

pgad096_Supplementary_Data

## Data Availability

The data underlying this article are available in the GitHub repository *Akkademia*, the translation demo in the Colab Notebook *Translating Akkadian to English using NLP*, and the supplementary information in the GitHub repository *AkkadiantoEnglish_NMT_SI*.

## Footnotes

1 The glyphs were generated with Cuneify, an online tool created by Steve Tinney and implemented in ORACC. The source code can also be found in GitHub.

2 These are available in JSON format thanks to the efforts of the Official Inscriptions of the Middle East in Antiquity (OIMEA) Munich Project of Karen Radner and Jamie Novotny, funded by the Alexander von Humboldt Foundation.

3 The more common spelling of the pronoun “I” is *a-na-ku*.
