# Supplementary material for: Translating Akkadian to English with neural machine translation
Source: PNAS Nexus. 2023 May 2;2(5):pgad096. doi: 10.1093/pnasnexus/pgad096 (PMC10153418; doi:10.1093/pnasnexus/pgad096)
Supplement: pgad096_Supplementary_Data [file pgad096_supplementary_data.zip › PNASNEXUS-PNASNEXUS-2022-00798-T-s15.pdf]

50 Sentences for testing the Akkadian-English Machine Translation

We selected the first five genres with the best attestations in the training data, and distributed the 50 sentences for this test accordingly:

**Text Genre**	**Number of Sentences**
Royal inscriptions	16
1 old Babylonian royal inscription	
Administrative letters	13
Legal transactions	10
Omens	6
2 astrological omens	
3 extispicy omens	
1 old Babylonian extispicy report	
Administrative records	5
Total of sentences for testing	50

We used Neo-Babylonian and Neo-Assyrian texts, which are well attested in the training data. Since the "astrological reports", "extispicy queries" and "extispicy reports" cite omens, we used astrological and extispicy omens, because in the Neo-Babylonian and Neo-Assyrian there are no more new texts in those genres. In the royal inscriptions and omens, we included Old Babylonian sentences. The language and the orthography in this period differ from the later periods.

The transliterations are presented as in their original publication. Because they come from different publications, they show different translations traditions. For the model to be used they have to be standardized for that purpose.

Royal Inscriptions (16 sentences)

Some royal inscriptions are similar, but not entirely equal.

S-1

The Embankment Inscription,
da Riva SANER 3, B5, Version 3.pp. 36-38 (1)

1 *a-na* ^{d}amar.utu en-*šú*
2 ^{d}ag-ibila-*ú-ṣur* <<ú-ṣur>> lugal ká.dingir.ra^{ki}
3 ti-ri-iṣ šu^{ii} ^{d}ag ù ^{d}amar.utu
4 kar a-gur-ri a-na ki-da-nu
5 bàd ká.dingir.ra^{ki}
6 ú-šá-as-ḫi-ir
7 kar *a-na* ^{d}amar.utu munus.sig$_5$-*šú qí-bi*

Translation
“To his lord Marduk, Nabopolassar, king of Babylon, designated by Nabû and Marduk, surrounded the wall of Babylon on the outside with an embankment of baked bricks. O embankment, speak well on his behalf Marduk.”

S-2.

The Arahtu Inscription
da Riva SANER 3, B4, Version 2, pp. 40-42 (1)

1 ^{d}⸢ag⸣-⸢ibila⸣-*ú*-[*ṣur*] lugal [ká.dingir.ra^{ki}]
2 *ti*-⸢*ri*⸣-⸢*iṣ*⸣ ⸢šu^{II}⸣ ^{d}ag ⸢*ù*⸣ [^{d}amar.utu]
3 ⸢*za*⸣-⸢*nin*⸣ é.⸢sag⸣.⸢íl⸣ *ù* ká.[dingir].ra^{ki}
4 dù^{eš} kar ^{íd}a-ra-aḫ-ti a-na ^{d}⸢amar⸣.utu ⸢en⸣-*šu*
5 kar *a-na* ^{d}amar.utu munus.sig5-*šu qí-bi*

Translation
“Nabopolassar, king of Babylon, designated by Nabû and Marduk, the provider of Esagil and Babylon, the builder of the embankment of the Arahtu canal, for his lord Marduk. O embankment, speak well of him to Marduk!”

S-3

The Etemenanki inscription, Babylon
da Riva SANER 3, B6, pp. 42-43 (1)

1 ^{d}*na-bi-um*-ibila-ùru
2 lugal ká.dingir.ra^{ki}
3 *šakkanakku* ^{kur}*šu-me-ri*

4 *u* uri^{ki} *mu-ki-in*
5 *iš-di* ma.da *a-na-ku*
6 *ì-nu-um* ^{d}amar.utu
7 en gal-*ú*
8 kur$^{(?)}$ *u ni-ši-im*
9 *a-na be-lim i-din-nam*
10 kur *a-a-bi-ja*
11 *ša-la-lam*
12 *iq-ba-am*
13 *ì-nu-mi-šu*
14 é.temen.an.ki
15 *e-eš-ši-iš*
16 *e-pu-uš-ma*
17 *a-na aš-ri-šu*
18 *ú-te-er*

Translation

"(1–5) Nabopolassar, king of Babylon, governor of Sumer and Akkad, who consolidates the land, I (am). (6–12) When the great lord Marduk gave me land and people to rule over, he ordered me to plunder my enemy's land. (13–18) At that time, I built anew the Etemenanki and I restored it to its place."

S-4

The Imgur-Enlil inscription, Babylon
da Riva SANER 3, C11/A 44, pp. 44-50 (1)

VA.5952
1 ^{d}*na-bi-um*-ibila-*ú-ṣu-úr*
2 lugal ká.dingir.raki
3 *ti-ri-iṣ qá-ti* ^{d}*na-bi-um*
4 *ù* ^{d}amar.utu *a-na-ku*
5 *im-gur*-^{d}en.líl
6 bàd *ra-bí-a-am*
7 *ša* ká.dingir.ra^{ki}
8 *ša ul-la-nu-ú-a*
9 *i-ni-šu i-qú-pu*
10 *in ki-gal-lim*$^{(!)}$ *re-eš-ti-im*
11 *ú-ki-in uš-ši-šu*
12 *in um-ma-nim di-ku-ut*$^{(!)}$ *ma-ti-ja*
13 *eš-ši-iš lu ab-ni-ma*
14 *a-na* im.límmu.ba ká.dingir.ra^{ki}
15 ⸢*lu ú-ša-às-ḫi-ir*
16 *ki-ma ša* u$_4$-*um ul-lu-tim*
17 *re-ši-šu lu ú-ul-lu*

18 bàd *a-na* ^{d}amar.utu en-*ja*
19 *da-mi-iq-ta ti-iz-*[*ka-ar*]

Translation
“(1–4) I am Nabopolassar, king of Babylon, designated by Nabû and Marduk. (5–19) (Regarding) Imgur-Enlil, the great wall of Babylon, which had become dilapidated and collapsed before my time, I firmly established its foundations on the original base. I built it anew with a levy of workers from my country, and I surrounded Babylon (with it) in the direction of the four winds. I raised its summit as it was in the old days. (20–21) O wall, speak good words (about me) to my lord Marduk!”

S-5

The Imgur-Enlil inscription, Babylon
da Riva SANER 3, C11/B, pp. 50-54 (1)

IM 80016
1 ^{d}*na-bi-um-*ibila-*ú-ṣur*
2 lugal ba-bi-lim^{ki}
3 *ti-ri-iṣ* šu^{II} dag ù ^{d}amar.utu *ana-ku*
4 *im-gur-*^{d}en-líl
5 bàd *ra-ba-a ša ba-bi-lim*^{ki}
6 *ul-tu* ⸢gú⸣ ^{íd}⸣*a-*⸢*ra*⸣*-aḫ-tim*
7 ús an.ta *šá* ká.gal ^{d}innana
8 *a-di* ús ki.ta *ša* ká.gal ^{d}⸢uraš⸣
9 *te-me-en-šu la-bi-ri*
10 ⸢*ú-ṣab*⸣*-bi-ma mit-ḫa-riš*
12 *a-na* ^{d}amar.utu en-*ja*
13 *lu-ú e-pu-uš*
14 bàd *a-na* ^{d}amar.utu *qí-bi* sig_5*-ti*

Translation
“(1–3) I am Nabopolassar, king of Babylon, designated by Nabû and Marduk. (4–13) (Regarding) Imgur-Enlil, the great wall of Babylon, from the bank of the Arahtu on the north side, that of the Ištar-Gate, to the south side, that of the Uraš-Gate, I inspected its old foundation and I built it in the same manner for my lord Marduk. (14) O wall, say good words to Marduk (Var. B4 and B5) on behalf of Nabopolassar.”

S-6

The E-edinna inscription, Sippar
da Riva SANER 3, C22, pp. 70-73 (1).

I

(1) ^{d}*na-bi-um*-ibila-*ú*-⸢*ṣu*⸣-[*úr*] / (2) lugal *da*-⸢*núm*⸣ / (3) lugal ká.dingir.ra^{k}[^{i}] / (4) lugal ma.da *šu-me-rí-im* / (4b) *ù ak-ka-di-i* / (5) ⸢*mu*⸣-*ki-in iš-di* ma.da / (6) *ru-ba-a-am na-*ʾ*-dam*/ (7) *ti-ri-iṣ qá-at* / (8) ^{d}*na-bi-um ù* ^{d}amar.utu / (9) *mi-gi-ir ša-aš-šu* / (10) *na-ra-am da-*⸢*a*⸣/ (11) *qar-ra-ad qar-ra-*[*de-e*] / (12) *ša* ^{d}*ìr-ra ra-šu-*⸢*ub*⸣-[*bu*] / (13) *ú-ša-ak-ši-du-šu ni-iz-*⸢*ma*⸣-⸢*su*⸣ / (14) *à-aš-ri-im ša-aḫ-ṭam* / (15) *mu-uš-te-*ʾ*-e-em sak-ke-e-em* / (16) *ša* dingir.dingir gal.gal / (17) lugal *ša ep-še-tu-šu* / (18) e-li lugal.lugal ad.ad-*šu* / (19) *šu-tu-qá a-na-ku* /

Translation
"(I 1–19) I am Nabopolassar, the mighty king, king of Babylon, king of Sumer and Akkad, who consolidates the land, reverent prince, designated by Nabû and Marduk, favourite of Šaššu, beloved of Aja, hero of heroes, the one (to) whom awe-inspiring Erra allowed to obtain his desire, the humble, the reverent, who is ever solicitous for the divine rituals of the great gods, the king whose deeds surpass those of the kings his ancestors. (I 20–II 4)

S-7

The E-edinna inscription, Sippar
da Riva SANER 3, C22, pp. 70-73 (1).

I
(20) *e-nu-ma ša-aš-šu* / (21) en *ra-bí-ù* / (22) *i-da-a-a il-li-ku-ma*

II
(1) [*su-ba-ru-um a*]-*na-ru* / (2) [*ù* ma.da *za-*ʾ*-e*]-*ri-ja* / (3) [*ú-te-ir-ru*] ⸢*a*⸣-*na* du$_6$ / (4) [*ù*] ⸢*ka*⸣-*ar-mu* / (5) ⸢*i*⸣-*nu-mi-šu* / (6) *a-na* ^{d}nin ud.kib.nun^{ki} / (7) *ru-ba-tim ṣi-ir-tim* gašan-*ja* / (8) é.edin.na é *ta-ap-šu-úḫ-ti-šu* / (9) *e-eš-ši-iš e-pu-uš-ma* / (10) *ki-ma* u$_4$-*mi-im ú-na-mi-ir* / (11) *a-na ša-at-tim* ^{d}nin ud.kib.nun^{ki} / (12) [*be-el*]-*tim šu-úr-bu-tim* / (13) [*e*]-⸢*ma*⸣ é *šu-a-ti* / (14) *uš-ta-ak-la-lu-ma* / (15) *ta-ra-am-mi-i-im qé-re-eb-ša* / (16) *ja-a-ši* ^{d}*na-bi-um*-ibila-*ú-ṣu-úr* / (17) lugal *za-ni-in-ki* / (18) *ki-ma* sig$_4$.sig$_4$ ud.kib.nun^{ki} / (19) *ù* ká.dingir.ra^{ki} / (20) *ku-un-na-am a-na ṣi-a-tim* / (21) *šar-ru*$_4$*-tim šu-ul-bi-ri-im* / (22) *a-na* u$_4$-*mi-im re-e-qú-tim*

Translation
When the great lord Šaššu came to my side, I kill[ed the Subarean (Assyrian) and turned] my ene[my's land] into tells and ruin heaps. (II 5–10) In those days, to Šarrat-Sippar, the supreme princess, my lady, I rebuilt the E-edinna, the temple of her rest, and I made it shine like daylight. (II 11–22) Therefore, O Šarrat-Sippar, greatest lady, whenever I complete this temple, and you establish your residence inside it, to me, Nabopolassar, the king your provider, like the bricks of Sippar and Babylon that are permanent forever, prolong my kingship to faroff days."

S-8

Brick inscription
da Riva SANER 3, B1, pp. 112-113 (1).

1 ^{d}u-gur-lugal-ùru lugal tin.tir^{ki}
2 mu-ud-diš è-sag-ìl ù è-zi-da
3 e-pi-iš da-am-qa-a-ti

Translation
"Neriglissar, the king of Babylon, the one who renovates Esagil and Ezida, the one who performs good actions."

S-9

Inscription fragment: restoration of a Šamaš(?) temple(?) or ziqqurat(?)
da Riva SANER 3, C021, pp. 138-140 (1).

5' [*i-n*]*a la-ba-ri* u$_{4}$-*um*
6' [*i*]*-ni-iš i-qú-up-ma*
7' [*zu*]*-un-nim ù ra-a-du*
8' *ra-bí-ú-tim*
9' *ú-na-as-su-ú i-ga-ru-ša*
10' *a-na te-di-iš-ti-ša*
11' ^{d}utu *da-a-a-nam ṣi-i-ri*
12 *ú-ša-ad-ka-an-ni li-ib-ba*
13' *li-bi-it-ta-ša*
14' *ú-uš-te-ši-ir-ma*
15' *ma-qí-it-ta-ša*
16' *ú-*[*uš-zi-iz-ma*]

Translation
"[...] in time past [it had] become weak, (and) had collapsed; heavy rain and storms had removed its walls. (10'–16') So Šamaš, the august judge, prompted me to renovate it. I put its bricks in order and I repaired its damage [...]."

S-10

Ziqqurrat inscription, Sippar
da Riva SANER 3, C022, pp. 140-143 (1).

I
1 ^{d}*nè-iri*$_{11}$*-gal*-lugal-*ú-ṣu-úr* lugal ká.dingir.ra^{ki}
2 *ru-ba-a-am na-a-dam*
3 *bi-ib-il li-ib-bi* ^{d}amar.utu
4 *iš-ša-ak-ku ṣi-i-ri*
5 *na-ra-am* ^{d}*na-bi-um*
6 *mu-uš-ta-lam a-ḫi-iz né-me-qí*
7 *ša a-na zi-in-na-a-ti* é.sag.íl
8 é.zi.da
9 ⌜ù⌝ ⌜é$^{(?)}$⌝.<<x>>$^{(?)}$.⌜mes$^{(?)}$⌝.⌜lam$^{(?)}$ *ma-ḫa-zi* dingir gal.gal /

10 [*ti-iṣ-mu-ru-um-ma*] ⸢*la*⸣ ⸢*na*⸣-*pa-ar-ka-a*

Translation
"(I 1–13) Neriglissar, king of Babylon, reverent prince, favourite of Marduk, august city-ruler, beloved of Nabû, the judicious one, full of wisdom, who incessantly [is mindful] about the support of Esagil, Ezida and ⸢Emeslam⸣(?), the cultic centres of the great gods (...).

S-11

Ziqqurrat inscription, Sippar
da Riva SANER 3, C022, pp. 140-143 (1).

2' *ṣu-lu-ul-šu* ⸢*ṭa*$^{(?)}$⸣-[*a-bu*]
3' *e-li um-ma-ni-ja* ⸢*it*$^{(?)}$⸣-[*ru-uṣ*]
4' *i-na di-i-nim ù bi-ri* ⸢^{d}⸣[utu$^{(?)}$ (x)]
5' *i-ša-ri-iš i-dá-ab-bu-*⸢*bu*⸣ [x x]
6' *pa-al-ḫi-iš at-ta-*ʾ-⸢*id*⸣-[*ma*]

Translation
(I 1'–6') (...) [he extend]ed(?) his
[favoura]ble(?) protection over my army (...) give me, [Šamaš], correct answers in pronouncements and divination, I give reverent attention.

S-12

Ziqqurrat inscription, Sippar
da Riva SANER 3, C022, pp. 140-143 (1).

I
7' *i-na šu-ru-ti* lugal-*ú-ti-ja ṭa-ab-ti*
II
1 *zi-qú-ra-at* ud.kib.[nun^{ki}]
2 *ša* lugal *ma-aḫ-ri i-p*[*ú-šu-ma*]
3 *zu-un-nu ù ra-a-*[*du*]
4 *ú-na-as-su-ú li-bi-it-t*[*u-ša*]
5 lugal *pa-nim ik-ši-ru-m*[*a*]
6 *li-bi-it-ta-ša ú-uš-te-*[*ši-ir-ma*]
7 *ma-qí-it-ta-šu ú-uš-*[*zi-iz-ma*]
8 22 kùš *i-pú-šu-ma la* ⸢*ú*⸣-[*šak-li-lu*]
9 *re-e-ša-*[*a-ša*]
10 *ja-ti e-*[*em-qa mu-ut-né-en-nu-ú*]
11 *ša pa-la-ḫa* dingir.din[gir *mu-du-ú*]
12 *i-na né-me-qí ša* ⸢^{d}⸣[*é-a*]
13 *iš-ru-*⸢*ka*⸣-[*am* (x)]
14 [*a*$^{(?)}$]-⸢*na*⸣ ⸢^{d}⸣⸢utu⸣ [

Translation
(I 7'–II 14) "In the beginning of my auspicious kingship, the ziqqurrat of Sippar, which a former king had bu[ilt but], rain and storm had removed [her] bricks, a previous king had repaired it, he had put her bricks in order, and [repa]ired its damage, he built (it) 22 cubits (high), but he did not [fin]ish it to its top, so I, the w[ise, the pious], who reveres the gods, [the expert one], with the skills that [Ea] granted me (...) for(?) Šamaš (...). (II 1'–2') (...) in pronouncement (...)."

S-13

The vase fragment V1
da Riva SANER 3, pp. 143-145 (1).

(1) 2 *qa* 1/2 ninda / (2) dnergal-lugal-ùru lugal tin.tir^{ki}

Translation
"2 qa (measure) 1/2 akalu (measure), Neriglissar, the king of Babylon."

S-14

The Etemenanki inscription, Babylon
da Riva SANER 3, C31, pp. 77-92 (1).

I
1 a-na ^{d}amar.utu en *ra-bí-ù*
2 ^{d}en.líl dingir.dingir *mu-uš-ta-ar-ḫa*
3 *a-ši-ir* $^{\langle d\rangle}$*i-*gi_4*-*gi_4
4 *sà-a-ni-iq* ^{d}a-nun-na-ki
5 *nu-úr* dingir.dingir *ab-bé-e-šu*
6 *à-ši-ib* é.sag.íl
7 en ká.dingir.ra^{ki} *be-lí-ja*

Translation
"(I 1–7) To Marduk, the great lord, the Enlil of the gods, the proud one, who directs the Igigi, who supervises the Anunnaki, the light of the gods his fathers, who dwells in Esagil, the lord of Babylon, my lord.

S-15

The Etemenanki inscription, Babylon
da Riva SANER 3, C31, pp. 77-92 (1).

I
8 ^{d}*na-bi-um*-ibila-*ú-ṣu-úr*
9 gìr.níta ká.dingir.ra^{ki}

10 lugal ma.da *šu-me-rí-im* / (10b) *ù ak-ka-di-im*
11 *ru-ba-a-am na-ʾ-dam*
12 *ti-ri-iṣ* <*qá-at*> ^{d}*na-bi-um* / (12b) *ù* ^{d}amar.utu
13 *à-aš-ru-um ša-aḫ-ṭam*
14 *ša pa-la-aḫ* dingir *ù* iš$_8$.dar
15 *li-it-mu-du ṣú-ru-uš-šu*
16 *za-ni-in* é.sag.íl *ù* é.zi.da
17 *mu-uš-te-ʾ-em sà-ak-ke-e*
18 ⸢*ša*⸣ dingir gal.gal *a-na-ku*

Translation
(I 8–18) "I am Nabopolassar, the šakkanakku of Babylon, the king of Sumer and Akkad, the pious prince, designated by Nabû and Marduk, the humble, the reverent, whose heart is well versed in the worship of god and goddess, the provider of Esagil and Ezida, who is ever solicitous for the divine rituals of the great gods."

S-16

Išme-Dagan of Isin
George, CUSAS 17, pp. 89-91 (2)

This is an Old Babylonian Text.

1. ^{d}*iš-me*-^{d}*da-g*[*an*]
2. *za-ni-in*
3. [*n*]*ippuru*(nibru)^{ki}
4. *tu-ku-ul-ti*
5. *urim*^{ki}
6. *a-še-er*
7. *eridu*(NUN)

Shaft col. I

8. *na-ṣi-i*[*r*]
9. *mi-i*
10. *unuk*(unug)^{ki}
11. *mu-ut eš*$_4$*-tar*$_2$
12. *e-lum*
13. ^{d}utu ì-si-in^{ki}
14. šar šu-me-ri-im
15. ì-lí
16. ⸢*ma*⸣*-at*
17. *wa-ri-im*
18. *ta-li-im*
19. ^{d}*en-líl*

Translation

"Išme-Dagan, provisioner of Nippur, mainstay of Ur, provider for Eridu, guardian of the rites of Uruk, sacred spouse of the goddess Ištar, sun of Isin, king of Sumer, god of the land of Wari'um, brother of the god Enlil

Administrative Letters (15 sentences)

For this case, we divided two texts in 10 sentences:

S-17

VAT 5650 (WVDOG 152 V 1000)
http://oracc.museum.upenn.edu/atae/X152001
o 1 IM ^{m}⸢BÀD⸣-*aš-šur*
o 2 *a-na maš-šur-re-ṣu-u-*⸢*a*⸣

Translation
Letter of Dūrī-Aššur to Aššur-rēṣūwa.

S-18

VAT 5650 (WVDOG 152 V 1000)
http://oracc.museum.upenn.edu/atae/X152001

o 3 *lu-u* DI-*mu ana* PAB-⸢*ia*⸣

Translation
May my brother be well.

S-19

VAT 5650 (WVDOG 152 V 1000)
http://oracc.museum.upenn.edu/atae/X152001

o 4 *ina* UGU GEŠTIN *šá te-ri-*⸢*iš*?⸣-[*u-ni*]
o 5 ⸢*la ta*⸣*-qa-bi ma-a ka-*
o 6 <*ka*>-[*ni*]*-ik ta-bi-ik*
b.e. 7 [UD]-⸢*mu*⸣ *šá ni-ib-zu*
r 1 *an-ni-u ta-mar-u-ni*

Translation
Regarding the wine that you requested: do not say, 'Is it sealed yet? Is it already stored?' On the day that you see this tablet,

S-20

VAT 5650 (WVDOG 152 V 1000)
http://oracc.museum.upenn.edu/atae/X152001

r 2 *šup-ra šúm-mu* GEŠTIN *a-na mì-*⸢*šil*⸣
r 3 *la-an-du-du a-na* 01*-en-iš* ⸢KI⸣.[MIN]
r 4 *la-din šúm-mu* DUG.ŠAB.MEŠ

r 5 *a-na* É-KAR GÁL-*še*

Translation
"[...] write to me (to tell me) whether I should measure out half of the wine or give the whole amount at once, (and moreover) whether there are jars for the trading post,"

S-21

VAT 5650 (WVDOG 152 V 1000)
http://oracc.museum.upenn.edu/atae/X152001

r 6 ⸢*šúm*⸣-*mu* 01-*ma šúm-mu ina* ½

Translation
«(specifically) whether the whole quantity or (only) half (is available).»

S-22

VAT 5650 (WVDOG 152 V 1000)
http://oracc.museum.upenn.edu/atae/X152001

r 7 GEŠTIN DÙG.GA *šu-u*

Translation
«The wine is good»

S-23

StAT 1 054 (8)
http://oracc.org/atae/P514500/
1 ⸢IM⸣ ^{m}15-I
o 2 ⸢IM⸣ ^{md}PA-NUMUN-AŠ
o 3 ⸢IM⸣ ^{m}*qí-bit-aš-šur*
o 4 ⸢IM⸣ ^{m}DI-*mu*-KUR-*aš-šur*
o 5 ⸢IM⸣ ^{m}PAB-*u-nu*
o 6 *a-na* ^{md}PA-MU-*iš-kun*
o 7 *a-na* ^{m}*aš-šur-kur-ub-šú-nu*

Translation
«Letter of Issār-naʾid, Letter of Nabû-zēru-iddina, Letter of Qibīt-Aššūr, Letter of Šulmu-māt-Aššūr (and) letter of Aḫūnu to Nabû-šumu-iškun (and) Aššūr-kurubšunu.»

S-24

StAT 1 054 (8)
http://oracc.org/atae/P514500/

o 8 *ina* UGU *de-ni šá da-ba-*⸢*ab-ni*⸣
o 9 *ṣi-a-né al-ka-né*

Translation
«On account of the lawsuit we have to conduct: come out, come here (to Assur)!»

S-25

StAT 1 054 (8)
http://oracc.org/atae/P514500/

o 10 DUMU URU.NINA.KI
b.e. 11 URU.*kal-ḫa-a-a*
b.e. 12 EN-*mu-ta-te-ni*
b.e. 13 *šú-nu de-nu*
r 14 *ina* IGI-*e-šú-nu*
r 15 *ni-du-ub-bu*

Translation
«A man from Nineveh (and) one from Kalḫu are the judges in our murder trial (literally, our "lords of deaths"). We have (already) conducted the lawsuit before them.»

S-26

StAT 1 054 (8)
http://oracc.org/atae/P514500/

r 16 *àna mì-ni ina* IGI URU.ŠÀ-URU-<*a-e*>
r 17 *a-e de-nu*
r 18 *ni-da-bu-ub*

Translation
«Why should we (also) conduct the lawsuit before a man from Libbi-āli?»

S-27

StAT 1 054 (8)
http://oracc.org/atae/P514500/

r 19 ^{m}15-I *maš-šur*-SUM-PAB.MEŠ
r 20 *an-na-ka e-gér-tú*

Translation
«Issār-naʾid (to) Aššūr-nādin-aḫḫē: The tablet is here.»

S-28

StAT 1 054 (8)
http://oracc.org/atae/P514500/

r 21 *ina* ŠU.2-*i-ka ṣa-bat*
r 22 *al-ka*

Translation
«Seize (it) in your own hand and come here!»

S-29

StAT 1 054 (8)
http://oracc.org/atae/P514500/

r 22 *mì-nu*
r 23 *šá de-en-ka-nu-ni*
t.e. 24 *du-ub-ba*

Translation
«Whatever is the situation with your lawsuit, conduct it!»

Legal Transactions (10 sentences)

S-30

BT 106
http://oracc.org/atae/P522826/

o 6 *ú-piš*-⸢*ma*⸣ ^{m}-*na-zi-i*
o 7 *ina* ŠÀ 13 MA.⸢NA⸣ URUDU.⸢MEŠ⸣
o 8 TA* IGI ⸢^{m}⸣*ka-šu-di* ⸢DUMU⸣ ^{m}DINGIR-SU
o 9 *il-qi*

Translation
«Nazî has contracted and acquired it for thirteen minas of copper from Kaššudu»

S-31

BT 118
http://oracc.org/atae/P522836/

o 7 *šum-ma la na-ṣa* ⸢*la i-din*⸣

Translation
«If he does not bring (him) and does not hand (him) over.»

S-32

BT 102
http://oracc.org/atae/P522822/

o 5 ^{m}*mar-di-i* DUMU-*šú*
o 6 *a-na šá-par-ti šá-kín*

Translation
«Mardî, his son, is placed as a pledge.»

S-33

BT 123
http://oracc.org/atae/P522839/

o 4 DUMU-*šú*
o 5 *a*-⸢*na*⸣ *šá-par-ti kam-mu*-[*us*]

Translation
«His son is placed as a pledge.»

S-34

BT 125
http://oracc.org/atae/P522841/

o 1 *ku-um* NA$_4$.KIŠIB *ṣu-pár-šú-nu*
o 2 *iš-kun-nu*

Translation
«Instead of their seals, they impressed their fingernails.»

S-35

BT 128
http://oracc.org/atae/P522844/

o 1 43 MA.NA URUDU.MEŠ SAG.DU
o 2 *ša* ^{m}*ti-ku-su*
o 3 *ina* IGI ^{m}*da-di-i*
o 4 *ina pu-u-ḫi it-ti-ši*

Translation
«Forty-three minas of copper, capital, belonging to Tikasu, at the disposal of Dādî. He has taken it as a loan.»

S-36

BT 126
http://oracc.org/atae/P522842/

o 8 [*la-qí*]-⸢*at*⸣ *tu-a-ru de-e-nu*
o 9 [DUG$_4$.DUG$_4$ *la*]*-a-šú man-nu ša* GIL*-u-ni*

Translation
«Any revocation, lawsuit or litigation is void. Whoever breaks the contract»

S-37

Schoyen 54
http://oracc.org/atae/P342655/

o 10 *a-na* DUMU.MÍ*-ti il-qe*
o 11 *kas-pu gam-mur ta-din*

Translation
«[She] has taken her as a daughter. The money is paid completely.»

S-38

al-Rafidan 17 10
http://oracc.org/atae/P522465/

r 7 DUMU.UŠ-*šú ra-bu-*(*u*)
r 8 [*ina ḫa-am-ri* ^{d}]IM *i-qa-lu*

Translation
«[he] shall burn his first-born son in the sacred precinct of Adad.»

S-39

Marqasu 03
http://oracc.org/atae/P522548/

o 3 ^{f}*ìr-tam-*^{d}IM GÉME ⸢ŠEŠ⸣-[*šú*]
o 4 *ú-piš-ma* ^{m}ARAD-*na-*<*na*>*-a-a*
o 5 TA 16 GÍN.MEŠ KÙ.BABBAR TA *pa-*⸢*an*⸣
o 6 ^{m}DI-EN-*áš-me il-qe*
o 7 *kas-pi ga-mur ta-din-ni*
o 8 GÉME *šu-a-te zar*$_4$*-pat la-qí-a-at*
o 9 *be-ni ṣib-tam a-na* 01 *me* UD-*me*
o 10 *sa-ar-ti a-na kàl* UD-*me*.MEŠ
o 11 *tu-a-ru de-ni* DUG$_4$.DUG$_4$ *la-áš-šú*

Translation
«Urdu-Nanāya has contracted and acquired Irtam-Adad, [his] (that is, Šulmu-bēli-lašme's) sister (tablet: brother), from Šulmu-bēli-lašme for sixteen shekels of silver. The money is paid completely. That female slave is purchased (and) acquired.»

Omens (5 sentences)

S-40

Astrology
Reiner, BPO 4, pp. 46-47 (3)

K.2080
DIŠ MUL SAG.ME.GAR *ina še-er-ti* [*ik-tu-un*] LUGAL.MEŠ KUR.MEŠ [SILIM.MEŠ]

Translation
«If Jupiter passes at the head of Venus: Akkad will be conquered with a strong weapon.»

S-41

Astrology
Reiner, BPO 2, pp. 48-49 (4)

Text VI (Sm 1267)
[DIŠ ^{mul}DIL.BAD *ina* SAR-*šá* ^{mul}MIN *ana* Š]À-*šú* TU-*ma* NU È-a DUMU LUGAL *ana* È AD-*šu*$_2$ TU-[ma AŠ.TE DIB-*bat*]

Translation
«If at Venus's rising the same star enters into it and does not come forth: the King's son will enter his father's house and seize the throne.»

S-42

Extispicy
Koch, AOAT 326, p. 236 (5).

25. *Multābiltu* Commentary 1 (Ms A = DT 49+)
I
26 be na *u* gír záḫ-meš-*ma ina* 150 zé gar-meš nun ina ur$_5$.[úš-*šú*]
27 zi-*aḫ-ma* man-*ma* gar-*an* dingir-meš-*u-a* [*ana*] kúr ⸢du.meš⸣

«If the Presence and the Path are destroyed and are placed to the left of the Gall Bladder: The prince will be removed from office and another installed, my gods will go over to the enemy.»

S-43

Extispicy
Koch, AOAT 326, p. 331 (5).

37. Combined Protases (K.4072)
III
29 [be sag šu.si kar-*i*]*m u ina* sag edin 15 u ^{giš}tukul gar-*ma* sag u igi

Translation
«If the top of the Finger is atrophied] and a Weapon is placed in the top of the right Plain of the Finger and points to the top of the Finger.»

S-44

Extispicy
Koch, AOAT 326, p. 381 (5).

58. Protases in Context (Ms B = K.220)
2' *ina* nu silim-*tim* tu-*ib* dumu *šip-ri*

Translation
«In an unfavourable extispicy: Admitting a messenger.»

S-45

Old Babylonian Extispicy Report
Koch Old babylonian Extispicy Reports, Fs. Walker, p. 133 (6).

4 *te-er-tum* ki.gub *i-šu* gír *i-šu* kal *i-šu*
5 silim *i-šu i-na* zag zé ^{giš}tukul *na-aḫ-bu-tum* gar
6 ze$_2$ zag *ki-na-at qú-tu-un* zé *ik-bi-ir*
7 *ku-bu-uš* mur du$_8$ mur zag *ta-li-il*
8 šu.si mur murub$_4$-*tum* zag *a-na* 2 du$_8$-*at*
9 *tal* ša$_3$ *re-šà i-šu*
10 14 *ti-ra-nu za-aq-ru*
11 1 udu *li-pí-it qá-ti a-na* alan *ḫa-mu-ra-bi*

Translation
«The extispicy: it has a Presence, it has a Path, it has a Strength, it has a Well-being. On the right side of the Gall Bladder there is a migrated Weapon, the Gall Bladder is firm on the right side, the Thin Part of the Gall Bladder is thick. The Lung's Cap is split, the Middle Finger of the Lung has two splits on the right. The Lintel of the entrails (the diaphragm) has its top, there are 14 Coils of the Colon; they are protuberant. A Sheep for the performance of extispicy concerning a statue of Hammurabi.»

Administrative Records (5 sentences)

S-46

CTN 1 11
http://oracc.org/atae/P370852/

o 1’ 05 *qa* [*ša*-É-02-*e*]
o 2’ ⸢05⸣ *qa* ⸢*ša*⸣-[É-^{m}*qi-qi-i*]

Translation
«5 litres, [the domestic servants]; 5 litres, the [qiqî-house] men»

S-47

CTN 1 p. 124, ND 10029
http://oracc.org/atae/P224663/

r 1 [0] ŠAB ⸢*ša*?⸣ GIŠ.Ì
r 2 [0] ^{md}PA-⸢ZU⸣
r 3 [0] ⸢SAG⸣.DU UR.MAḪ LÚ.SUKKAL
r 4 [0] ^{m}mu-še-zib-^{d}MAŠ

Translation
«a jar of oil Nabû-le’i lion's head (rhyton), the vizier of Mušezib-Ninurta.»

S-48

CTN 1 pl. 45, ND 10026
http://oracc.org/atae/P370874/

r 5 PAB 21 ANŠE 4 BÁN [05] *qa ša* MAN-*pu-ḫi*
r 6 PAB-*ma* 76 ANŠE 6 BÁN 08 *qa* GEŠTIN-MEŠ KÚ

Translation
«Total 21 homers 4 seah 5 qa, of the substitute king. Total 76 homers 6 seah 8 qa, wine consumption.»

S-49

CTN 2 130
http://oracc.org/atae/P428851/

r 1 PAB 34 ANŠE.KUR-MEŠ
r 2 03 ANŠE.GÌR.NUN.NA

r 3 08 ANŠE.NÍTA-MEŠ
r 4 02 GUD-MEŠ

Translation
«In all 34 horses, 3 mules, 8 donkeys, 2 oxen.»

S-50

StaT 2 097
http://oracc.org/atae/P336905/

o 1 02 ANŠE 1 BÁN 05 *qa* GIG
o 2 *ša* ^{m}*qur-di*-^{d}ME.ME
o 3 *ina* IGI ^{m}PAB-*ba-ni*
o 4 A ^{m}*gab-bu-ana-aš-šur*
o 5 *ina* ITI.BARAG (SUM) *šúm-mu*
o 6 *là* SUM-*ni pu-tú-ḫu*
b.e. 7 É-*šú i-na-ši*
b.e. 8 ^{m}NIM-UŠ-DU EN-ŠU.2-ME
r 1 ITI.ŠE *lim-mu* ^{m}NU-MAN-E
r 2 IGI ^{m}*aš-šur*-SUM-PAB-⸢MEŠ⸣
r 3 IGI ^{m}AŠ-DI-*mu*-PAB

Translation
«Two homers one seah 5 liters of wheat belonging to Qurdi-Gula, at the disposal of Ahu-bani, son of Gabbu-ana-Aššur. He shall (pay) in month Nisan (I). If he does not pay, he shall bear the responsibility for his house. ... is the guarantor. Month Adar (XII), eponym year of Ṣalam-šarri-iqbi. Witness Aššur-nadin-ahhe. Witness Aššur-šallim-ahi.»

References

1. Da Riva R (2008) *The Neo-Babylonian Royal Inscriptions* (Ugarit-Verlag, Münster).
2. George AR, ed (2011) *Cuneiform Royal Inscriptions and Related Texts in the Schøyen Collection* (CDL Press, Bethesda, Maryland).
3. Reiner E, Pingree D (2005) *Babylonian Planetary Omens: Part Four* (Styx, Groningen).
4. Reiner E, Pingree D (1981) *Babylonian Planetary Omens: Part Two. Enūma Anu Enlil, Tablets 50-51* (Undena Publ, Malibu).
5. Koch US (2005) *Secrets of Extispicy. The Chapter Multābiltu of the Babylonian Extispicy Series and Niṣirti bārûti Texts mainly from Aššurbanipal's Library* (Ugarit-Verlag, Münster).
6. Koch-Westenholz US (2002) in *Mining the Archives. Festschrift for Christopher Walker on the occasion of his 60th birthday*, ed Wunsch C. (Islet, Dresden), pp 131–145.
