# Supplementary material for: Translating Akkadian to English with neural machine translation
Source: PNAS Nexus. 2023 May 2;2(5):pgad096. doi: 10.1093/pnasnexus/pgad096 (PMC10153418; doi:10.1093/pnasnexus/pgad096)
Supplement: pgad096_Supplementary_Data [file pgad096_supplementary_data.zip › PNASNEXUS-PNASNEXUS-2022-00798-T-s16.pdf]

5 Texts Test

The translations for S-1 and S-2 are available in ORACC. Transliteration and translation for S-3, S-4 and S-5 were made by the authors for this test. The texts are not published but the photos are accessible on CDLI and on the website of the British Museum.

S-1 Legal Transaction.

This text is published only with a German translation.

BATSH 6 31
http://oracc.org/atae/P527156/

o 1 NA$_4$.KIŠIB ^{m}*ḫu-zi-*⸢*ri*⸣
very faint sealing
o 2 DUMU *ma-di*-TE-DINGIR.MEŠ
o 3 EN É SUM-*an*
o 4 É *pi-rík-tú ša*
o 5 É ^{m}U.GUR-I
o 6 *ina* ŠÀ-*bi ṣi-in-a-ni*
o 7 *ú-piš-ma* ^{m}DI-*mu*-MAN
o 8 *ina* ŠÀ-*bi* 05 GÍN.MEŠ KÙ.BABBAR
o 9 TA* IGI ^{m}*ḫu-zi-ri*
o 10 TI-*qe kas-pu gam-mur*
o 11 *ta-din* É *za-rip*
o 12 *laq-qe tu-a-ru*
o 13 *de-e-ni* DUG$_4$.DUG$_4$
o 14 *la-a-šú man-nu*
o 15 *ša* GIL-*u-ni*
o 16 kas-pu a-na 03-te
b.e. uninscribed
r 1 *a-na* EN.MEŠ [GUR-*ra*]
r 2 *ina de-ni-šú* ⸢DUG$_4$⸣.[DUG$_4$-*ma*]
r 3 *la* (*i*)*-laq-*⸢*qe*⸣
r 4 ⸢IGI⸣ ^{md}UTU-MU-[AŠ]
r 5 ⸢IGI⸣ ^{m}A.10-⸢KALAG⸣
r 6 ⸢IGI⸣ ^{m}AŠ-PAB.[MEŠ]
r 7 ⸢IGI⸣ ^{m}*sa-kip*-[x-x]
r 8 IGI ^{m}⸢KAM⸣-*eš*-[x-x]
r 9 IGI ^{m}EN-[x-x]
r 10 IGI ^{md}⸢PA⸣-PAB-[x]
r 11 IGI ^{md}⸢*nusku*⸣-MAN-[PAB]
empty space

r 12 ITI.ZÍZ UD-14-⸢KÁM⸣
r 13 *lim-me* ^{m}EN-KUR-*u*-[*a*]
l.e. 1 20 GIŠ.ÙR.MEŠ [*ina*? ŠÀ?-*bi*?]

Translation

«Seal of Huziri, son of Adi-sukki-ilāni, the owner selling the house. It is the blocked house by the house of Nergal-naʾid, there are small animals in it. Šulmu-šarri has contracted and acquired it for 5 shekel of silver from Huziri. The silver is completely paid. The house is purchased (and) acquired. Any revocation, lawsuit or litigation is void. Whoever breaks the (contract) shall pay threefold to the owners. Should he litigate in the lawsuit, he will not succeed. Witness: Šamaš-šumu-iddina. Witness: Apla-dad-daʾʾin. Witness: Iddin-ahhe. Witness: Sakip-x. Witness: Ereš-x. Witness: Bel-x. Witness: Nabu-ahu-x. Witness: Nusku-šarru-uṣur. Monat Šabāṭu (XI), 14th day. Eponym year of Bel-šadua. 20 beams in its interior?»

S-2 Legal Transaction.

This text is published only with a German translation. For a separate test, it is split into smaller sentences which correspond to the division of text lines in the original document This was done to compare the results between long and short sentences in NMT.

BATSH 6 110
http://oracc.org/atae/P527235/

o 1 *de-e-nu ša mú-ar-bi-is* LÚ.*sar-ten* 1
o 2 *maš-šur*-DINGIR-*a-a* LÚ.SUKKAL *dan-nu*
o 3 *e-mi-du-u-ni* UD-15-KÁM* *ša* ITI.SIG$_4$
o 4 ^{m}*ra-ḫi-mi*-DINGIR LÚ.EN-GIŠ.GIGIR
o 5 *a-na* ^{m}A.10-DINGIR-*a-a* LÚ.SIPA *ú-ba-la*
o 6 TA* ^{m}U.GUR-MAN-PAB *ú-ka-na*
o 7 *šùm-ma la na-ṣa la ú-ki-ni*
b.e. 8 NÍG.MEŠ UDU *a-di lid-da-ni-šú-nu*
b.e. 9 08 ZI.MEŠ *a-di sa-ár-ti-šú-nu*
r 1 ^{m}*ra-ḫi-mi*-DINGIR *a-na* ^{m}U.GUR-MAN-⸢PAB⸣
r 2 SUM-*an* ITI.GU$_4$ UD-28-⸢KÁM⸣
r 3 *lim-mu* ^{m}⸢*mil-ki*⸣-*ra-mu*
r 4 IGI ^{m}[*ra*]-*pa-ia-u* IGI ^{m}EN-DÙG.GA
r 5 IGI ^{m}15-E ⸢IGI⸣ [^{m}]⸢*su*⸣-*u-a*
r 6 IGI ^{md}PA-*še-zib* IGI ^{m}*na-zi-i*
t.e. 7 IGI ^{md}PA-*rém-a-ni*

Translation

«(Result of) a court case wich Uarbis, the chief judge, (and) Ashur-ila'i, the great vizier, have imposed. On the 15th of the month Sivan (III) Rahimi-il, the chariot owner, will bring Apladad-ilāya, the sheperd. He will together with Nergal-šarru-uṣur confirms (it). If he does not bringt (him) and not confirm (it), the property, (namely) the sheep together with their kids (and) eight

persons along with the fine for them Rahimi-il will hand (them) to Nergal-šarru-uṣur. Month of Iyyar (II), the 28th day, Eponym year of Milki-ramu. Witness: Rapʾ-Iau. Witness: Bēl-ṭab. Witness: Issar-iqbi. Witness: Suʾa. Witness: Nabû-šezib. Witness: Nazî. Witness: Nabû-remanni.»

S-3 Astrological Omen.

K.8900 + K.8914
https://cdli.ucla.edu/search/archival_view.php?ObjectID=P238822

r 1 DIŠ ^{mul}*ṣal-bat-a-nu* KAN$_5$ UN-MEŠ ⸢*ša*$_2$⸣$^?$ ⸢SU$^?$.GU$_7$⸣$^?$ ⸢IGI-MEŠ⸣ NINDA ⸢*nap*⸣-*ša*$_2$
⸢GU$_7$⸣-MEŠ ŠA$_3$ KUR DUG$_3$-*ma*
r 2a BURU$_{14}$ KUR SI.SA$_2$ MAŠ$_2$.ANŠE ^{d}GIR$_3$ SI.SA$_2$

Translation
«If Mars is becomes dark, the people that have seen famine will eat plentiful bread, the inner of the land will become good, the harvest of the land will prosper.»

S-4 Oracular question.

K.20148
https://cdli.ucla.edu/search/archival_view.php?ObjectID=P418623

o 1’ x x x [...]
o 2’ *ša*$_2$ SAR-MEŠ-⸢*šu*$_2$⸣ *u*[*t*$^?$/*p*[*i*$^?$ ...]
o 3’ *qu-rad* KUR$_2$-*šu*$_2$ *na-a*[*š*$_2$ ^{giš}TUKUL ...]
o 4 *u*$_2$-*saḫ-ḫar-šu*$_2$-*nu*-⸢*ti*⸣ [...]
o 5’ u$_2$-ṣi KUR$_2$-šu$_2$ iš-x [...]
o 6’ ^{giš}TUKUL-MEŠ-*šu*$_2$ UGU ^{g}[^{iš}TUKUL-MEŠ KUR$_2$-*šu*$_2$ ŠEŠ-MEŠ-*u*$_2$ ...]
o 7’ *taḫ-ta-a* x [...]
o 8’ x [...]
r 1’ ^{m}*aš-šur*-D[U$_3$-IBILA LUGAL GAL LUGAL *dan-nu* LUGAL ŠU$_2$ LUGAL ^{kur}AN.ŠAR$_2$^{ki}]
r 2’ DUMU ^{m}AN.[ŠAR$_2$-ŠEŠ-ŠUM$_2$-*na* LUGAL ŠU$_2$ LUGAL ^{kur}AN.ŠAR$_2$^{ki}]
r 3’ [DUM]U ^{md}30-P[AP-MEŠ-SU LUGAL ŠU$_2$ LUGAL ^{kur}AN.ŠAR$_2$^{ki}-ma]

Translation
«... warrior of his enemy, the weapon-bearer ... he surround them ... he expulses his enemy ... will his weapons prevail over his enemy’s weapons? ... defeat? ... Aššur-bani-apli, great king, strong king, king of the world, king of Assyrian, son of Esarhaddon, king of the world, King of Assyria, son of Sennacherib, king of the world, king of Assyria.»

S-5 Unknown (Erra Epic?).

BM.38754
https://www.britishmuseum.org/collection/object/W_1880-1112-638

I

1’ [...] x *ik-ki-bi*
2’ [...] x GE$_6$ *u im-ma*
3’ [...] x-*ra* DINGIR *ik-ki-bi*
4’ [... *i*]*š-te-ne*$_2$*-ʾe ha-ba-lu*
5’ [...-*i*]*k*$^?$ *u*$_2$*-pa-ra-ru ga-nu-nu*
6’ [...] x ⸢u$_2$⸣-ra-⸢as-sa⸣-*bi ba-nu-ti*
7’ [...] *i-na-a-ri dan-nu-ti*
8’ [...] *u*$_2$*-šam-qat*$_2$ *ru-be*$_2$*-e*
9’ [...]-⸢*um*⸣-*bil* KI.SIKIL-MEŠ
10’ [...] x *ut*$^?$*-tu-ru* KAŠ
11’ [...] x *ma-ḫar*
12’ [...] x [(x)]

II

1’ x [...]
2’ x [...]

Translation
«... of the taboo ... the night and the heat ... god of the taboo ... he sought out the wrongdoing ... he dissolved the storeroom ... he smote the good ... he stroke the strength ... he brings to fall the prince ... the girl ... the beer ... the first one.»
