# Supplementary material for: Translating Akkadian to English with neural machine translation
Source: PNAS Nexus. 2023 May 2;2(5):pgad096. doi: 10.1093/pnasnexus/pgad096 (PMC10153418; doi:10.1093/pnasnexus/pgad096)
Supplement: pgad096_Supplementary_Data [file pgad096_supplementary_data.zip › PNASNEXUS-PNASNEXUS-2022-00798-T-s17.docx]

1

2 Supplementary Information for

3 **Translating Akkadian to English with Neural Machine Translation**

4 **Gai Gutherz, Shai Gordin, Luis Sáenz, Omer Levy, Jonathan Berant**

5 **Gai Gutherz**

6 **E-mail:** [**gaigutherz@gmail.com**](mailto:gaigutherz@gmail.com)

7 **Shai Gordin**

8 **E-mail:** [**shaigo@ariel.ac.il**](mailto:shaigo@ariel.ac.il)

# 9 This PDF file includes:

10 Supplementary text

11 Fig. S1

12 Tables S1 to S3

13 Legends for Dataset S1 to S16

14 SI References

# 15 Other supplementary materials for this manuscript include the following:

16 Datasets S1 to S16

# 17 Supporting Information Text

18 **Training Details**

19 Tables S1 and S2 show the BLEU4 scores for different values of max tokens and learning rates for C2E and for T2E respectively.

20 We computed these numbers as part of the process of selecting hyperparameters. The best result in each table is marked in

21 bold.

**Table S1. C2E: BLEU4 score for different hyper parameters**

| **max tokens**  **learning rate** | **4000** | **8000** |
| --- | --- | --- |
| **0.05** | 35.82 | 32.28 |
| **0.1** | **36.52** | 35.63 |

**Table S2. T2E: BLEU4 score for different hyper parameters**

| **max tokens**  **learning rate** | **4000** | **8000** |
| --- | --- | --- |
| **0.05** | 36.64 | 32.21 |
| **0.1** | **37.47** | 34.77 |

22 **Training Corpus**

23 For this research, we tried to include every corpus in ORACC that have both transliteration and full translation. The corpora

24 used are the following:

## 25 1. The Royal Inscriptions of the Neo-Assyrian Period (RINAP)

26 • RINAP 1: These royal inscriptions cover the reigns of Tiglath-pileser III (744-727 BCE) and Shalmaneser V (726-722

27 BCE)

28 • RINAP 2: Sargon II (721–705 BC)

29 • RINAP 3: Sennacherib (704-681 BCE)

30 • RINAP 4: Esarhaddon (680-669 BCE)

31 • RINAP 5: Ashurbanipal (668-ca. 631 BCE) and Successors Aššur-etel-il¯ani (ca. 631–627/626 BC), and Sîn-šarra-

32 iškun (627/626–612 BC)

## 33 2. The Royal Inscriptions of Assyria online (RIAo)

34 3. **The Royal Inscriptions of Babylonia online (RIBo) Project**

35 • Babylon 2: Second Dynasty of Isin (1157-1026 BC)

36 • Babylon 3: Second Dynasty of the Sealand (1025-1005 BC)

37 • Babylon 4: Bazi Dynasty (1004-985 BC)

38 • Babylon 5: Elamite Dynasty (984-979 BC)

39 • Babylon 6: Uncertain Dynasties (978-626 BC)

40 • Babylon 7: Neo-Babylonian Dynasty (625-539 BC)

41 • Babylon 8: Akkadian inscriptions of the Persian Period (538-330 BC), especially the now-famous ”Cyrus Cylinder”

42 • Babylon 10: Seleucid era (305-64 BC) official inscriptions written in Akkadian, especially the ”Antiochus (Borsippa)

43 Cylinder”

## 44 4. State Archives of Assyria Online (SAAo)

45 • SAA 01: The Correspondence of Sargon II, Part I: Letters from Assyria and the West

46 • SAA 02: Neo-Assyrian Treaties and Loyalty Oaths

47 • SAA 03: Court Poetry and Literary Miscellanea

48 • SAA 04: Queries to the Sungod: Divination and Politics in Sargonid Assyria

49 • SAA 05: The Correspondence of Sargon II, Part II: Letters from the Northern and Northeastern Provinces

50 • SAA 06: Legal Transactions of the Royal Court of Nineveh, Part I: Tiglath-Pileser III through Esarhaddon

51 • SAA 07: Imperial Administrative Records, Part I: Palace and Temple Administration

52 • SAA 08: Astrological Reports to Assyrian Kings

53 • SAA 09: Assyrian Prophecies

54 • SAA 10: Letters from Assyrian and Babylonian Scholars 54

55 • SAA 11: Imperial Administrative Records, Part II: Provincial and Militar Administration

56 • SAA 12: Grants, Decres and Gifts of the Neo-Assyrian Period

57 • SAA 13: Letters from Assyrian and Babylonian Priests to Kings Esarhaddon and Assurbanipal

58 • SAA 14: Legal Transactions of the Royal Court of Nineveh, Part II: Assurbanipal Through Sin-šarru-iškun

59 • SAA 15: The Correspondence of Sargon II, Part III: Letters from Babylonia and the Eastern Provinces

60 • SAA 16: The Political Correspondence of Esarhaddon

61 • SAA 17: The Neo-Babylonian Correspondence of Sargon and Sennacherib

62 • SAA 18: The Babylonian Correspondence of Esarhaddon and Letters to Assurbanipal and Sin-šarru-iškun from

63 Northern and Central Babylonia

64 • SAA 19: The Correspondence of Tiglath-Pileser III and Sargon II from Calah / Nimrud

65 • SAA 20: Assyrian Royal Rituals and Cultic Texts

66 • SAA 21: The Correspondence of Assurbanipal, Part I: Letters from Assyria, Babylonia, and Vassal States

67 • SAAS 2: Assyrian Eponym List

68 5. **The Inscriptions of Suhu online Project (Suhu)**

# 69 Distribution of the corpora according to genres

70 It is important to note that “text” is not to be understood as a unique text. A royal inscription, for example, could be attested

71 in many copies, i.e. many texts. For the text typologies see the website of [Archival Texts of the Assyrian Empire](http://oracc.museum.upenn.edu/atae/browsetextsbygenre/index.html) (ATAE) project.

72

**Table S3. Percentage of each text genre used in this research.**

| **Text Genre** | **Number of Texts** | **Percentage** |
| --- | --- | --- |
| Royal inscriptions | 2997 | 37.20% |
| Administrative Letters | 2003 | 24.86% |
| Legal Transactions | 829 | 10.29% |
| Astrological Reports | 567 | 7.04% |
| Administrative Records | 453 | 5.62% |
| Scholarly Letters | 389 | 4.83% |
| Extispicy Queries | 278 | 3.45% |
| Priestly Letters | 210 | 2.61% |
| Extispicy Reports | 76 | 0.94% |
| Grants | 67 | 0.83% |
| Royal Rituals | 55 | 0.68% |
| Literary Works | 52 | 0.65% |
| Eponym Lists | 23 | 0.29% |
| Treaties | 15 | 0.19% |
| Decrees | 14 | 0.17% |
| Votive Donations | 12 | 0.15% |
| Prophecies | 11 | 0.14% |
| Appointments | 4 | 0.05% |
| Gifts | 1 | 0.01% |
| Total | 8056 | 100% |

# 73 Description of supplementary information datasets

74 There are three types of SI file: txt files which contain the output of the model’s results; csv files which contain qualitative

75 assessment on a selection of the model’s test results and the additional human vs. machine case-studies; bibliographic

76 information for the texts used for the additional human vs. machine case-studies.

77 In the .txt files which include the model’s outputs, the source or input is preceded by an S, the original human translation is

78 T, and the output machine translation is D.

79 All datasets are available at the Github of the [Digital Pasts Lab](https://github.com/DigitalPasts/Akkadian_English_article_SI).

80

81

82

83

84

85

86

87

88

89

90

91

92

93

94

95

96

97

98

99

100

101

102

103

104

105

106

107

108

109

110

111

112

113

114

115

116

**SI Dataset S1 (best_run_test_T2E.txt)**

Output of the best model trained on T2E task on the test data.

**SI Dataset S2 (best_run_test_C2E.txt)**

Output of the best model trained on C2E task on the test data.

**SI Dataset S3 (sample_T2E.txt)**

Instances taken from Dataset S1 for random sampling.

**SI Dataset S4 (sample_assesment_T2E.csv)**

Table with the assessment of the instances used for random sampling in Dataset S3.

**SI Dataset S5 (sample_C2E.txt)**

Instances taken from Dataset S2 for random sampling.

**SI Dataset S6 (sample_assesment_C2E.csv)**

Table with the assessment of the instances used for random sampling in Dataset S5.

**SI Dataset S7 (HvsM_50_T2E.txt)**

The output of the T2E task on the 50 additional sentences for testing. They were extracted from published editions, see Dataset S15.

**SI Dataset S8 (HvsM_50_assesment_T2E.csv)**

Table with the assessment of the instances used in Dataset S7.

**SI Dataset S9 (HvsM_50_C2E.txt)**

The output of the C2E task on the 50 additional sentences for testing. These are the same texts as in Dataset S7.

**SI Dataset S10 (HvsM_50_assesment_C2E.csv)**

Table with the assessment of the instances used in Dataset S9.

**SI Dataset S11 (HvsM_5_T2E.txt)**

5 sentences in transliteration to test the T2E task. The first two sentences are only published in German, and the other three sentences were extracted from unpublished tablets. The texts of these were treated as single sentences, but in the case of the second sentence, we also split the text into sentences (2.1-2.19). For the bibliographical information of these texts, see Dataset S16

**SI Dataset S12 (HvsM_5_assesment_T2E.csv)**

Table with the assessment of the instances used in Dataset S11.

**SI Dataset S13 (HvsM_5_C2E.txt)**

The same 5 sentences as in Dataset S11 but in Unicode cuneiform to test the C2E task.

**SI Dataset S14 (HvsM_5_assesment_C2E.csv)**

Table with the assessment of the instances used in Dataset S13.

**SI Dataset S15 (50_sentences_bibliography.pdf.)**

Bibliographic references, published editions and translations of the 50 texts used for testing the model in Datasets S7 and S9.

**SI Dataset S16 (5_texts.pdf)**

Bibliographic reference of the 5 texts used for testing the models in Datasets S11 and S13.

117

118

119

# References

1. Frahm E (2011) *Babylonian and Assyrian text commentaries*, Guides to the Mesopotamian textual record. (Ugarit-Verl., Münster).


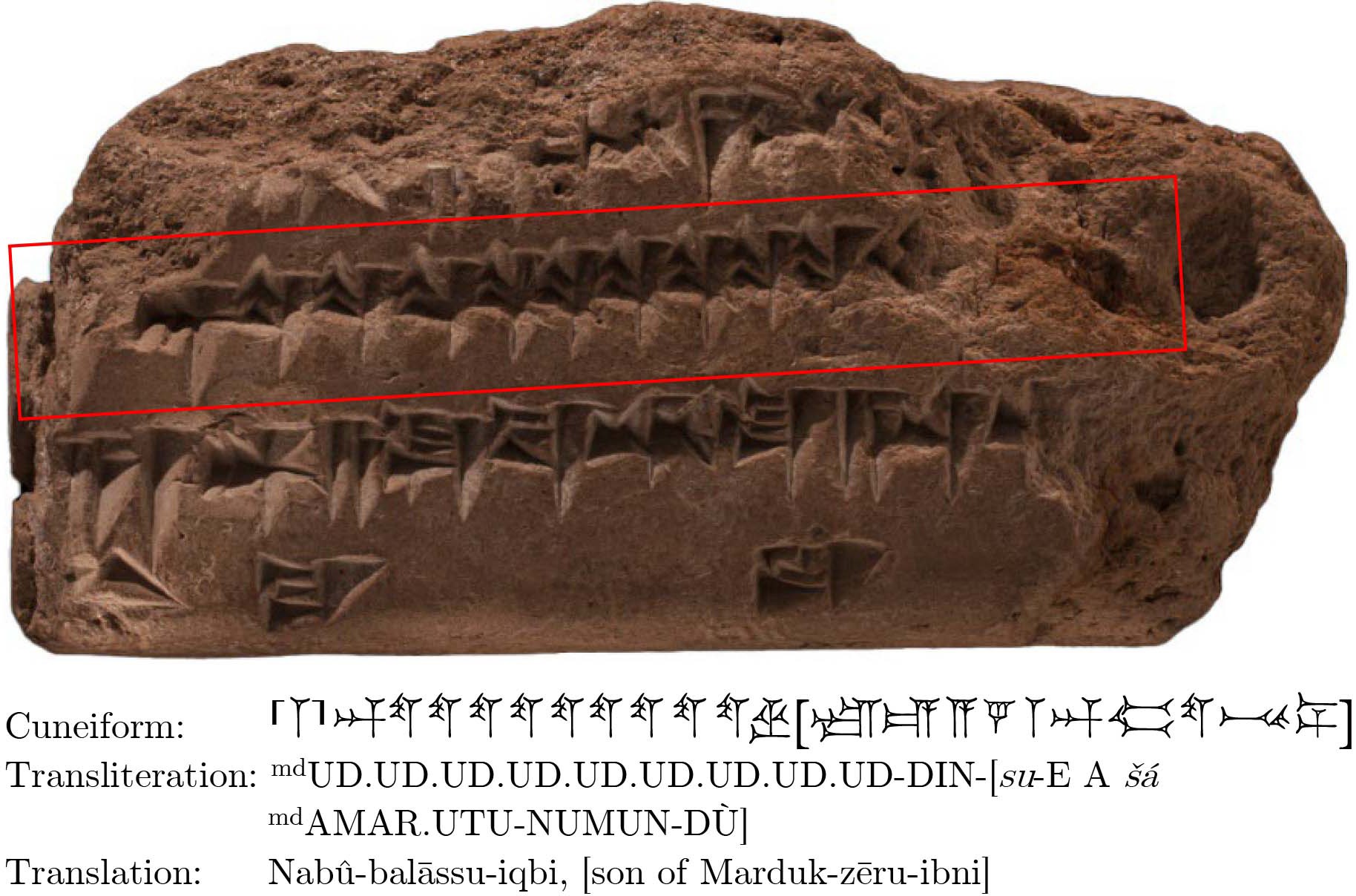


**Fig. S1.** Tablet [DT.84](https://cdli.ucla.edu/search/search_results.php?SearchMode=Text&ObjectID=P238789) from the Library of Ashurbanipal in Nineveh (© The Trustees of the British Museum). The obverse contains a commentary on extispicy. The reverse (photo) contains a colophon mentioning the author of the tablet ([1](#_bookmark0)), whose name is written with the logograms
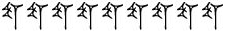
“UD.UD.UD.UD.UD.UD.UD.UD.UD”. In such cases, the name of the sign in capitals is used to designate an uncertain sign value.
